# Myeloid-Derived Suppressor Cells and Radiotherapy: Regulation and Clinical Implications

**DOI:** 10.7150/jca.132006

**Published:** 2026-04-08

**Authors:** Lixian Yang, Ke Wang, Wenjie Zeng, Yanwei Lu, Hailong Sheng, Hanchu Xiong, Haibo Zhang

**Affiliations:** Cancer Center, Department of Radiation Oncology, Zhejiang Provincial People's Hospital (Affiliated People's Hospital), Hangzhou Medical College, Hangzhou, Zhejiang, China.

**Keywords:** MDSCs, RT, immunosuppression, radioresistance

## Abstract

Radiotherapy (RT) serves as a critical treatment modality for cancer, which not only destroys local tumor cells, but also exerts the "double-edged sword" impact on the immune system. While triggering anti-tumor immune response, RT also induces a great number of immunosuppressive cells, including a predominant cell population, myeloid-derived suppressor cells (MDSCs). Notably, the RT-induced MDSCs play a vital role in radioresistance. To enhance the efficacy of RT, numerous preclinical studies have elucidated the mechanisms by which RT modulates MDSCs, and evaluated the MDSC-targeted strategies to sensitize RT. Moreover, some clinical studies have explored drugs involved in MDSC-targeting to enhance the effectiveness of RT. However, though generally tolerable, the clinical benefits of combining these agents with RT are limited. Lacking specificity of MDSC-targeted agents is a major concern. Furthermore, several fundamental challenges complicate the investigation of MDSCs and their interplay with RT. These include the lack of specific biomarkers for MDSCs, their dynamic behavior during RT, differences between murine and human immune systems, the complexity of standard-of-care regimens, and the involvement of other immunosuppressive cell populations. Future investigations need to keep focusing on uncovering the features of MDSCs and their roles in RT, so that we can develop specific therapies to target MDSCs individually. This review covers the complicated regulatory network between RT and MDSCs, as well as current preclinical and clinical strategies targeting MDSC to sensitize RT, aiming to provide some insights and directions for cancer therapy.

## 1. Introduction

RT stands as a cornerstone of cancer treatment, with over 70% of cancer patients requiring this intervention during their clinical course 1. Driven by rapid technological innovation, RT has evolved from two-dimensional techniques to advanced conformal and intensity-modulated modalities characterized by high-dose-rate delivery 2,3. Causing direct and indirect DNA damage on tumor cells is the fundamental cytotoxic mechanism of RT and thus facilitates local tumor control 4. Additionally, recent investigations underscore RT as a powerful "double-edged sword" for systemic immunity. On the positive side, RT predominantly functions as an “*in-situ* vaccine” by inducing immunogenic cell death (ICD) of tumor cells. ICD triggers tumor antigen exposure and DAMPs release, and DAMPs act as endogenous adjuvants to boost adaptive immunity 5. RT also promotes antigen presentation and recognition by upregulating the expression of MHC class I molecule on tumor cells 6. Furthermore, RT enhances innate immune response by activating the cyclic GMP-AMP synthase (cGAS)-stimulator of interferon genes (STING)-interferon (IFN) signaling pathway 7. As a result, RT-induced immune response enhances local tumor control and even induce abscopal effect, a phenomenon which local RT results in the remission of non-irradiated tumor sites 8. Consequently, the capacity of RT to stimulate positive immune responses is exciting and needs extensive exploration.

However, the pro-tumorigenic impact of RT on the immune system is equally potent. RT recruits a variety of immunosuppressive cells, upregulates immune checkpoint molecules like programmed death-ligand 1 (PD-L1), and fosters a hijacked and immunosuppressive microenvironment. This not only restricts the effectiveness of RT but might also lead to tumor metastasis 9. Among the immunosuppressive cells, MDSCs, tumor-associated macrophages (TAMs) and regulatory T cells (Tregs) are regarded as critical mediators in radioresistance. Elucidating their respective roles in mediating resistance and developing targeted strategies to counteract their effects are of great significance. However, in viewing of the complexity of their interplay with RT, this review primarily focuses on the bidirectional regulation between RT and MDSCs, while also briefly discussing the involvement of other immunosuppressive cell populations. MDSCs are a heterogeneous population of immature myeloid cells induced in pathological conditions like cancer, primarily categorized into polymorphonuclear MDSCs (PMN-MDSCs) and monocytic MDSCs (M-MDSCs) 9. MDSCs potently suppress effector immune cells, such as T cells, by depleting essential amino acids, producing reactive oxygen/nitrogen species (ROS/RNS), and secreting inhibitory cytokines. Beyond immune suppression, MDSCs also directly contribute to non-immunological and tumor-promoting processes, including angiogenesis, epithelial-mesenchymal transition (EMT), and pre-metastatic niche formation 10. Notably, MDSCs share origins and functionality with other myeloid cells like M2-type TAMs, and they can interconvert, collectively forming a potent "myeloid immunosuppressive axis" within the tumor immune microenvironment 11,12. The remarkable success of immunotherapies, such as chimeric antigen receptor T cell (CAR-T), PD-1/PD-L1, and CTLA-4 inhibitors, have sparked immense interest in combining them with RT. However, although some clinical trials show improved survival, the overall outcomes remain sub-optimal 13. A crucial reason is likely that RT concurrently induces a powerful MDSC-dominated immunosuppressive network, which undermines the efficacy of RT.

Considering that MDSCs almost exclusively accumulate in pathological conditions and exert crucial tumor-promoting effect, a comprehensive understanding of the complex interplay between RT and MDSCs is imperative. This review first briefly introduces the main characteristics of RT which involves MDSCs regulation. Then, it describes the development, molecular features and general function of MDSCs. We summarize the mechanisms by which RT induces MDSCs and MDSCs contribute to radioresistance, as well as the preclinical and MDSCs-targeted strategies to sensitize RT. Additionally, many clinical investigations involved RT and MDSCs-targeted agents have been reported and summarized in this review. We hope to provide researchers and clinical workers with insights into a new target that may sensitize RT or to overcome radioresistance.

## 2. RT and Immunity

Since the RT regimen serves as a critical determinant of therapeutic effect and subsequent immune modulation, it is essential to first delineate the primary RT modalities.

### 2.1 Types of RT

Owing to their distinct biological impacts, different RT modalities exert differential effects on the immune system 14,15, and uniquely modulate MDSCs (Table 3). In short, the modulation of MDSCs by RT is not uniform, but is determined by radiation types, fractionation, dose, and tumor models. Data from limited parallel-controlled studies suggest that hypofractionated RT (HypoRT) may be more prone to increasing MDSCs than single-dose irradiation 16, whereas ablative RT appears more effectively depleting them compared to daily fractionation 17, though these observations warrant cautious interpretation.

The classification of RT is multifaceted and can be classified by several ways, predominantly across four dimensions (Table 1). It reflects a fundamental shift of RT from old modalities to modern ones. In summary, RT is no longer a one-size-fits-all approach, but evolves into a precise and personalized treatment regimen. The current techniques focus on maximizing tumor targeting while sparing healthy tissues, so as to obtain the greatest clinical benefits.

### 2.2 Immunomodulatory effects of RT

**Figure 1:**
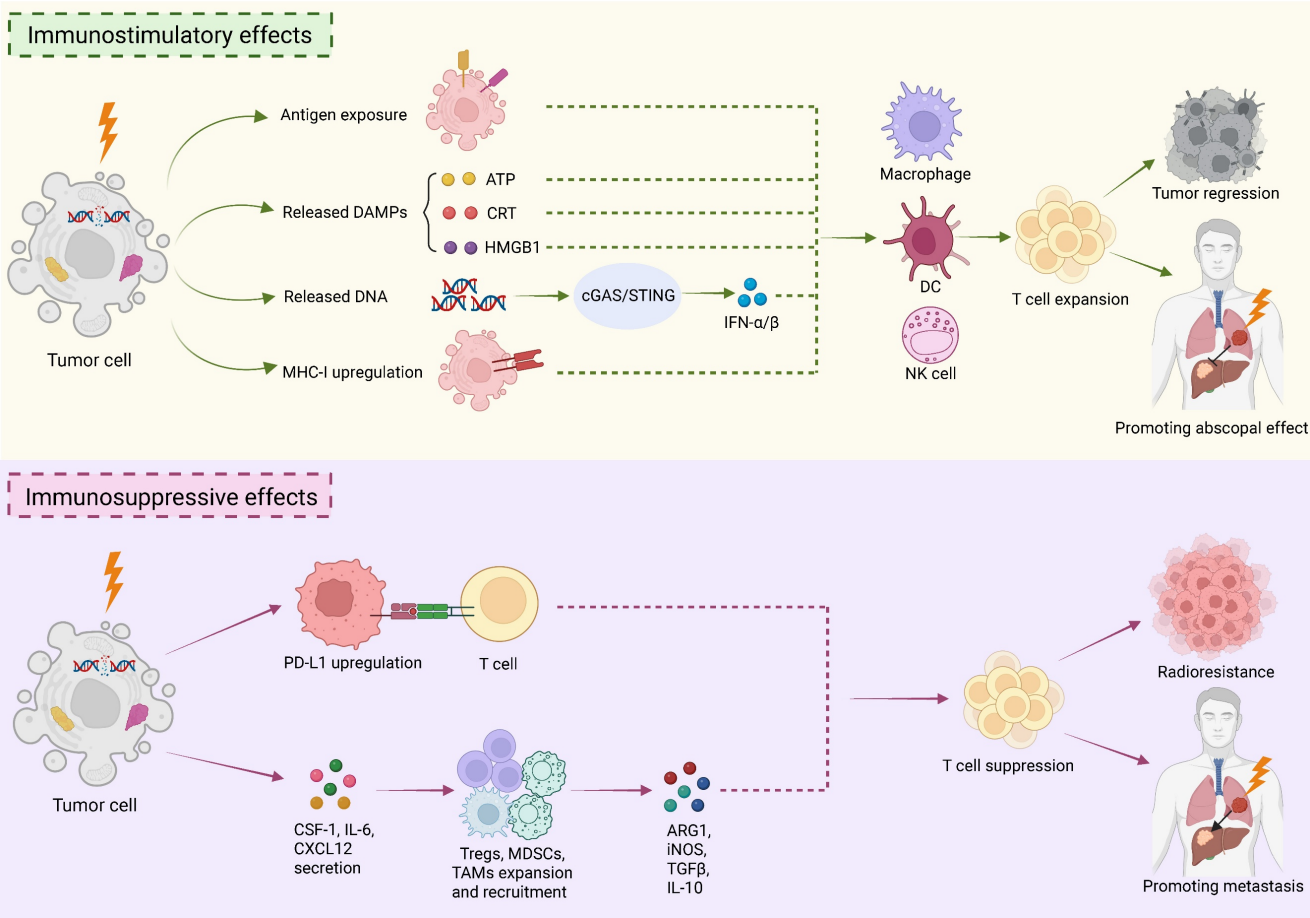
** Immunomodulatory effects of RT.** RT exerts a dual role in tumor immunity. It can both stimulate a systemic anti-tumor immune response and reprogram the immunosuppressive microenvironment. Upper: RT induces ICD of tumor cells, which exposes tumor antigens, releases DAMPs, such as ATP, CRT, HMGB1, and DNA. The released DNA activates the cGAS/STING signaling pathway, which promotes type I interferon secretion, such as IFN-α/β. Additionally, RT upregulates MHC-I expression on tumor cells. The mechanisms above collectively recruit and activate DCs, macrophages, and NK cells, which promote the expansion and anti-tumor effects of T cells. The immunostimulatory effect of RT not only enhances local tumor control but also promotes abscopal effect. Lower: RT also has immunosuppressive effects. RT upregulates the expression of PD-L1 on tumor cells and certain immune cells. Moreover, RT activates tumor cells to produce a variety of cytokines, such as CSF-1, IL-6, and CXCL-12, which in turn recruit and expand the key immunosuppressive cells, including Tregs, MDSCs and TAMs. These cells then release immunosuppressive molecules like Arg1, iNOS, TGF-β, and IL-10. Together, these mechanisms counteract the efficacy of RT, leading to radioresistance and even promote metastasis. The figure was created with BioRender.

It is now well recognized that RT impacts the therapeutic outcomes of tumors not only by directly killing tumor cells, but also by bidirectionally modulating immune system (Fig.1) 9. Therefore, it is very significant to generally overview the relationship between RT and immunity, which will offer a better understanding of the role of MDSCs, one core member of immune system, in RT (Fig. 1).

The key mechanism by which RT promotes anti-tumor immunity is the "*in situ* vaccine" effect. Specifically, RT-induced cell damage enhances the exposure of tumor-associated antigens, which facilitate immune recognition and thus activates adaptive anti-tumor immune responses 27. At the same time, RT-induced ICD causes the release of DAMPs, which serve as vaccine adjuvants to strengthen immune responses. Pivotal DAMPs comprise calreticulin (CRT) exposed on tumor cell surface, extracellular ATP, and high mobility group box 1 (HMGB1) 5. CRT serves as a "eat me" signal, which attracts antigen-presenting cells (APCs) to phagocytize tumor cells. ATP act as a "find-me" signal, which recruits APCs to the tumor site. HMGB1 promotes dendritic cells (DCs) maturation and the secretion of pro-inflammatory cytokines by binding to Toll-like receptor 4 (TLR4) on DCs. Additionally, radiation-induced DNA release activates the crucial cGAS-STING immune signaling pathway. The pathway activation triggers a signaling cascade that drives the production of type I interferons α/β (IFN-α/β) and various pro-inflammatory cytokines 7. IFNs serve as a pivotal bridge, connecting innate and adaptive immunity. They enhance the cross-presentation capacity of DCs, directly activate natural killer (NK) cells, and promote T cell survival and memory formation 28. Additionally, RT upregulates MHC class I molecule expression on tumor cells, thereby strengthening antigen presentation and recognition 6. Furthermore, radiation accelerates the release of various chemokines that recruit T lymphocytes, NK cells, and macrophages to the tumor sites. These immune cells collectively make up an anti-tumor army conducted by cytotoxic T lymphocytes, substantially augmenting RT efficacy 9,29. The generated memory T cells can subsequently seek out and eliminate tumor cells at distant locations, ultimately inducing the abscopal effect 9,29. Therefore, RT triggers anti-tumor immunity mainly through acting as “*in situ* vaccine” and induce the release of DAMPs to strengthen adaptive immunity, as well as augmenting innate immune response by activating cGAS-STING pathway (Fig. 1).

Unfortunately, RT also markedly induces an immunosuppressive microenvironment (Fig.1). It limits the RT efficacy, keeps the abscopal effect rare, and may even result in metastasis. The major mechanism is the upregulation of PD-L1 on tumor and immune cells. It directly suppresses the function of effector T cells and thereby there is a rationale for combining RT with immune checkpoint inhibitors (ICIs) 9. RT also stimulates tumor cells to secret cytokines, such as CSF-1, IL-6, and CXCL12. These cytokines recruit and expand immunosuppressive cells, including Tregs, MDSCs (see below), and TAMs in the tumor microenvironment (TME) 9. The immunosuppressive cells then secrete suppressive molecules such as arginase 1 (Arg1), inducible nitric oxide synthase (iNOS), TGF-β, and IL-10, which expand Tregs or directly inhibit cytotoxic T lymphocyte function 9. Overcoming this RT-induced immunosuppression is a critical research area. The remarkable success of ICIs in cancer immunotherapy has stimulated extensive exploration of combination strategies with RT 30,31. While results of some clinical trials are promising 32,33, many other clinical trials have not met their endpoints 34-36. Reasons for failure are multifaceted, including tumor PD-L1 status and immune cell infiltration 9. Powerful immunosuppressive cell populations probably represent another key factor, and thus more in-depth research is essential.

As discussed, RT regimens are complicated and can be classified predominantly across four dimensions. While RT elicits anti-tumor immune response, it also paradoxically promotes pro-tumor immunity (Fig.1). MDSCs represent a key immunosuppressive population that limits the efficacy of RT and may even results in metastasis. Therefore, gaining a deep insight into the relationship of MDSCs and RT is critically important.

## 3. MDSCs

This section delineates the fundamental characteristics of MDSCs (Table 2) to facilitate their laboratory identification, and covers their development, differentiation, and recruitment to the TME. We provide a comprehensive synthesis of the well-recognized tumor-promoting functions of MDSCs, encompassing both immunological and non-immunological mechanisms. The systematic overview of MDSCs lays foundation for understanding their role in radioresistance.

### 3.1 Main characteristics and definition of MDSCs

MDSCs originate from altered myelopoiesis of hematopoietic stem cells (HSCs) under pathological conditions. Normally, HSCs give rise to immature myeloid cells (IMCs) and IMCs further differentiate into monocytes and granulocytes. Monocytes then develop into macrophages and DCs. Granulocytes differentiate into neutrophils, basophils, and eosinophils. However, in pathologies such as chronic inflammation, cancer, or autoimmune disease, IMCs differentiation is diverted, which results in the formation of pathological MDSCs. According to morphology and phenotype, MDSCs are mainly divided into two major categories: PMN-MDSCs and M-MDSCs 10. PMN-MDSCs and M-MDSCs share numerous phenotypic and morphologic features with neutrophils and monocytes respectively. Additionally, PMN-MDSCs and M-MDSCs seems play distinct roles in tumor progression. In general, PMN-MDSCs take up a larger population in the TME, whereas M-MDSCs demonstrate stronger immunosuppressive activity and greater plasticity. Specifically, PMN-MDSCs are prone to subdue T cell function by producing TGF-βand ROS, as well as via an antigen-dependent manner. In contrast, M-MDSCs predominantly suppress T cells by disturbing L-arginine metabolism. M-MDSCs highly express Arg1 and iNOS, both of which consume L-arginine in the TME, resulting in T cell energy by starvation. iNOS also generates nitric oxide (NO) to dampen immune response. Additionally, M-MDSCs are pretty tend to differentiate into TAMs, another cell type with potent tumor-promoting functions 10.

Beyond PMN-MDSCs and M-MDSCs, there are two additional distinct subsets that have been identified. They include early-stage MDSCs (eMDSCs), which are more immature progenitor cells, and fibrocystic MDSCs (F-MDSCs), a unique human population, emerged in nonmalignant disorders 37. Compared to the normally differentiated myeloid cells, MDSCs have distinct characteristics. They have an immature phenotype and morphology, and also have weak phagocytic activity and potent immunosuppressive functions 10,37.

At the molecular phenotypic level, PMN-MDSCs and M-MDSCs are very similar to normal neutrophils and monocytes, and there are only a few surface markers that show differential expression. Mouse MDSCs were originally characterized by the co-expression of CD11b (integrin αM) and Gr-1 38. Gr-1 is an epitope that exist both in Ly-6G and Ly-6C molecules but do not present in humans. Moreover, the Gr-1 (RB6-8C5) antibody binds with high affinity to Ly-6G molecule but lower affinity to Ly-6C. Therefore, Gr-1 (RB6-8C5) antibody has been widely used for MDSCs depletion in infected or tumor-bearing mice 39. PMN-MDSCs are defined by the surface markers CD11b⁺Ly6G⁺Ly6C^low^, whereas M-MDSCs are characterized as CD11b⁺Ly6G⁻Ly6C^high^ in mice 40. However, there are no specific cell surface markers that could distinguish classical neutrophils from PMN-MDSCs or classical monocytes from M-MDSCs in mice. Therefore, MDSCs must be identified by their function of suppressing other immune cells.

In humans, proposed markers are CD15⁺/CD66b⁺CD14⁻LOX1⁺ or CD15⁺/CD66b⁺CD14⁻CD84⁺ for PMN-MDSCs. For human M-MDSCs, markers include CD14⁺/CD66b⁻CXCR1⁺ or CD14⁺/CD66b⁻CD84⁺ 40. Importantly, human PMN-MDSCs can be purified from neutrophils by their lower density. As to M-MDSCs, they can be distinguished from monocytes by detecting the expression of MHC class II (e.g. human leukocyte antigen HLA-DR), although it is not sufficient 40. The phenotypical markers and experimental approaches that can distinguish MDSCs from normal granulocytes and monocytes are briefly summarized (Table 2). Notably, it is necessary to detect the core functions of MDSCs, which include expressing immunosuppressive molecules, such as Arg1 and ROS, and inhibiting T cell function 41. In summary, the gold-standard identification of MDSCs relies on both the detection of a set of proposed makers and the assessment of their core suppressive functions. Moreover, the lack of specific surface markers probably impedes the elucidation of the characteristics and function of MDSCs in tumor progression, as well as their clinical translation.

### 3.2 Development, differentiation and recruitment of MDSCs

The ontogeny of MDSCs in malignancy is traditionally elucidated by a two-phase model. The initial phase involves the tumor-driven expansion of IMCs in the bone marrow and spleen. The second phase is their activation into immunosuppressive MDSCs by pro-inflammatory cytokines in peripheral tissues 42. Recently, this framework has been refined into a four-step model, encompassing myelopoiesis, systemic mobilization, recruitment to the tumor site, and intratumoral retention (Steps I - IV) 43.

Myelopoiesis, the process of blood cell formation, originates from HSCs in the bone marrow. HSC-derived common myeloid progenitors (CMPs) give rise to granulocyte-macrophage progenitors (GMPs) 10. Under the stimulus of granulocyte-macrophage colony-stimulating factor (GM-CSF), granulocyte (G)-CSF, macrophage (M)-CSF, and stem cell factor (SCF), GMPs subsequently differentiate into macrophage/dendritic cell progenitors (MDPs) and myeloblasts (MBs) 44. However, this finely tuned process is subverted in cancer. Myeloid cells undergo pathological activation due to their prolonged exposure to growth factors and inflammatory mediators. Key growth factors, such as GM-CSF, G-CSF, and M-CSF, drive the abnormal proliferation of myeloid progenitors in the bone marrow 45. Persistent inflammatory signals (e.g. IL-1β, IL-6, S100 calcium-binding protein A8/A9 (S100A8/A9)), along with vascular endothelial growth factor (VEGF), and chemokines (e.g., CCL2, CXCL5, CXCL12), collectively confer potent immunosuppressive functions for MDSCs 37. The JAK-STAT signaling pathway, involving STAT1, STAT3, STAT5, and STAT6, plays a central role. This pathway promotes MDSCs survival while arresting maturation by inhibiting regulators like interferon regulatory factor 8 (IRF8). As a result, it prompts MDSCs to upregulate the expression of key immunosuppressive molecules, mainly including TGF-β, IL-10, Arg1, iNOS and ROS 37. Simultaneously, the NF-κB pathway, as a central hub of inflammatory signaling, synergizes with JAK-STAT to amplify inflammatory responses and sustain MDSCs survival 37. These MDSCs are recruited to the TME by tumor-associated C-C and C-X-C motif chemokines and their receptors, such as CCL2/CCR2, CXCLs-CXCR1/2 and CCL5/ CCR5 ligand axes, as well as numerous other cytokines (e.g., CSFs, PGE2, IL-4, IL-6, IL-10, IL-1β, TNF-α, S100A8/A9, TGF-β) 10. A key characteristic of MDSCs is their remarkable plasticity. Within the TME, M-MDSCs can further differentiate into TAMs, and these TAMs typically exhibit an immunosuppressive M2-like phenotype 12. Additionally, it is hypothesized that N2 classification of tumor-associated neutrophils (TANs) is partly derived from MDSCs 46. Furthermore, MDSCs can also arise from the reprogramming of existing differentiated monocytes and granulocytes 10. Summarily, normal myeloid cells are driven by various cytokines and signaling pathways, which lead to diversion from their normal differentiation into immunosuppressive MDSCs. Moreover, the TME can reprogram even the terminally differentiated neutrophils and macrophages into MDSCs, which shows the strong ability of tumors to co-opt normal immune cells.

### 3.3 Tumor-promoting function of MDSCs

**Figure 2:**
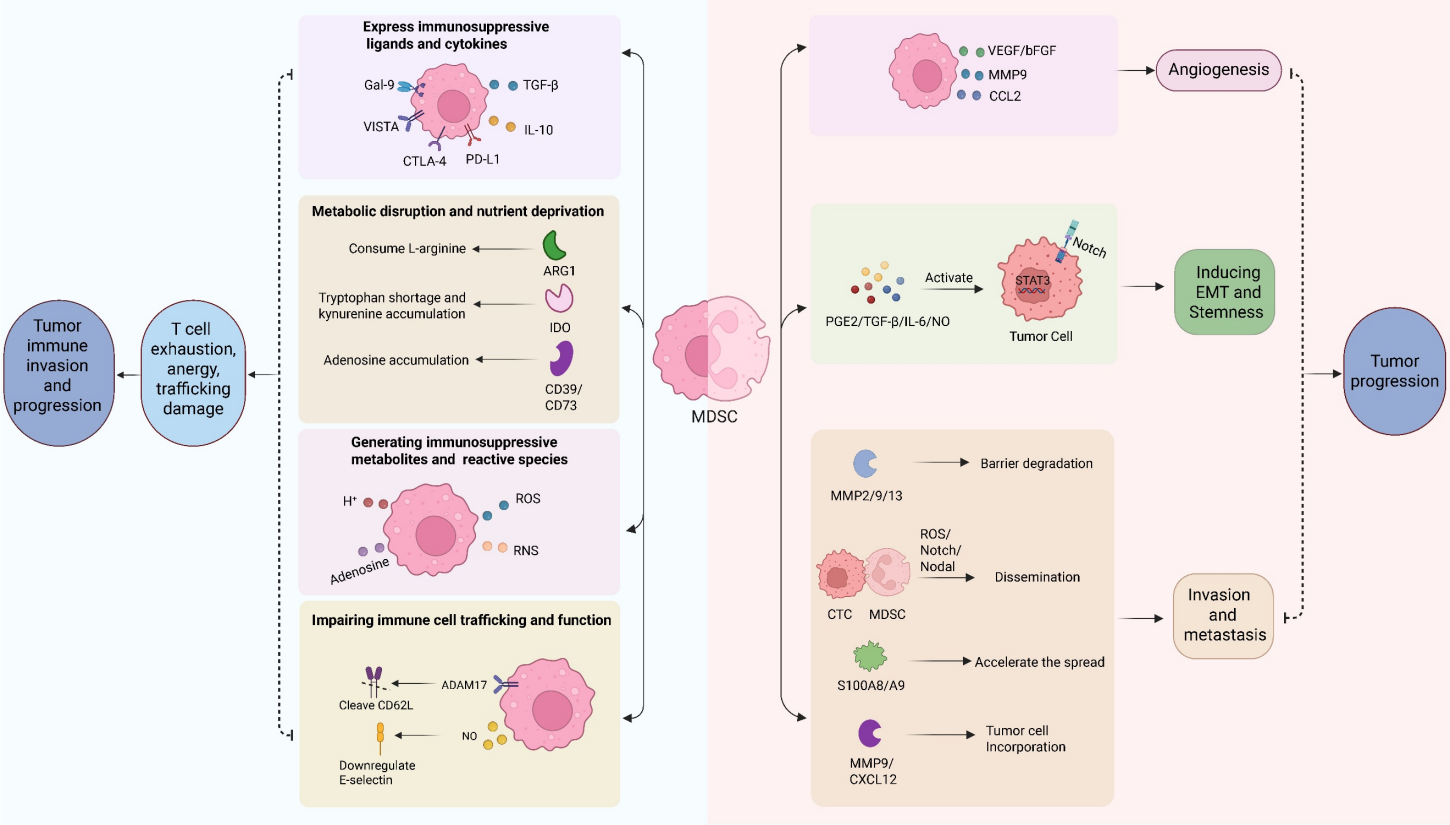
** Tumor-promoting mechanisms of MDSCs.** MDSCs, like M-MDSCs and PMN-MDSCs subsets, are driving tumor progression via two mechanisms: immunosuppression and direct pro-tumor facilitation. Immunosuppressive, MDSCs employs multiple strategies to suppress anti-tumor immunity: (1) expressing immunomodulatory ligands like PD-L1, CTLA-4, VISTA and Gal-9, and expressing immunosuppressive cytokines like IL-10 and TGF-β; (2) disrupting metabolism via enzymes like Arg1 (depleting L-argine), IDO (producing kynurenine), and CD39/CD73 (generating adenosine) to impair T-cell function; (3) Generating immunosuppressive metabolites like adenosine and H^+^, and reactive species like NO and ROS; (4) impeding immune cell trafficking by cleaving CD62L via ADAM17 and reducing E-selectin expression. Collectively, these actions cause T-cell exhaustion, anergy, and poor recruitment, which promotes immune evasion. Beyond immunosuppression, MDSCs also directly promotes tumor progression from several aspects: (1) stimulating angiogenesis through factors such as VEGF, bFGF, MMP9, and CCL2; (2) enhancing tumor aggressiveness and stemness by activating STAT3/NOTCH pathways via secreted factors like PGE2, TGF-β, IL-6, NO; (3) facilitating tumor metastasis by secreting protease (MMP2/9/13) to degrade barriers, forming clusters with CTCs via ROS/Notch/Nodal signaling, accelerating the spread of tumor cells via chemoattractants S100A8/A9, and supporting tumor cells incorporation into metastatic sites through MMP9 and CXCL12.

MDSCs contribute to tumor progression via various mechanisms, which encompass both the suppression of anti-tumor immunity and non-immunological pathways (Fig. 2).

#### 3.3.1 Immune suppression

MDSCs primarily drive tumor progression by orchestrating a tolerogenic TME via multiple inhibitory strategies (Fig. 2).

##### 3.3.1.1 Expressing immunosuppressive ligands and cytokines

MDSCs express various surface molecules to directly impair effector cell function. A primary mediator is PD-L1. Numerous studies have demonstrated that MDSCs upregulated PD-L1 expression on itself and other cells, like B cells, to engage PD-1 on T cells and NK cells, inducing their exhaustion 47. Hypoxia and soluble factors in TME facilitate to drive PD-L1 expression 48,49. MDSCs can also express other checkpoint molecules like cytotoxic T lymphocyte-associated antigen 4 (CTLA-4), V-domain Ig-containing suppressor of T-cell activation (VISTA), and galectin-9 (Gal-9) 10. These checkpoint molecules further reduce T cell activity.

Additionally, MDSCs serve as a dominant reservoir of the immunosuppressive cytokines IL-10 and TGF-β in the tumor-bearing host. The production of IL-10 by MDSCs is regulated by a variety of factors in the TME. They include hypoxia, Toll-like receptor (TLR) ligands like lipopolysaccharide (LPS), transmembrane TNF-α, and specific tumor-derived signals such as semaphorin 4D, as well as exosomes from glioma stem cells 50. The MDSC-derived IL-10 is a significant mechanism for polarizing macrophages to M2 phenotype, inducing Tregs, and suppressing DCs 50. Blocking IL-10 signaling can reverse the effects and improve anti-tumor immunity. Similarly, MDSCs also produce TGF-β, which is another well-known immunosuppressive cytokine. The production of TGF-β can be context-dependent and is regulated by transmembrane TNF-α, ribosomal protein S19 and semaphorin 4D 9. TGF-β is involved in the suppression of T, B, NK, and NKT cells, with the latter usually via membrane-bound form 51. Moreover, TGF-β is crucial for the induction of Tregs by MDSCs 52. Beyond direct immune suppression, MDSC-derived TGF-β plays a critical role in promoting tumor metastasis by inducing EMT in tumors 53.

##### 3.3.1.2 Metabolic disruption and nutrient deprivation

Metabolic reprogramming is fundamental for MDSC-mediated immunosuppression. By highly expressing the cationic amino acid transporter (CAT-2B) and Arg1, MDSCs deplete L- arginine from the TME 54,55. Consequently, arginine deficiency impairs T cell receptor signaling and halts T cell proliferation. MDSCs also sequester cysteine, another essential amino acid for T cell activation 56. Additionally, indoleamine 2,3-dioxygenase (IDO) catalyzes the catabolism of tryptophan in MDSCs. Tryptophan shortage and the accumulation of its metabolite, kynurenine, cause T cell anergy and support Tregs development 57.

##### 3.3.1.3 Generating immunosuppressive metabolites and reactive species

MDSCs exploit the adenosine pathway as a crucial immunosuppressive mechanism in the TME. MDSCs convert extracellular ATP to immunosuppressive adenosine by expressing ectoenzymes CD39 and CD73 on their surface. Moreover, TGF-β has been reported to reinforce this process via the mTOR-HIF-1α pathway 58. The accumulated adenosine binds to A2A and A2B receptors on immune cells, which strongly suppresses the function of T and NK cells 59. Furthermore, adenosine signaling, especially via the A2B receptor, creates a positive feedback loop that expands MDSCs populations and facilitate them to secret suppressive molecules, such as IL-10 60. Additionally, MDSCs acidify the TME by expressing H^+^ channels, which hampers cytotoxic lymphocyte activity and thus promotes tumor cells survival and mobility 61.

A series of ROS/RNS have been demonstrated to suppress anti-tumor immunity. MDSCs produce NO via upregulating iNOS expression, which hampers T cells activation by blocking IL-2R signaling and T cell receptors (TCRs). They also nitrate chemokines like CCL2, which reduces the recruitment of immune cells into tumors 10. MDSCs, mainly PMN-MDSCs, utilize ROS, which is produced by the NADPH oxidase isoform 2 (NOX-2), to suppress anti-tumor immunity. Once forming the peroxynitrite with NO, ROS disrupts T cell function by nitrating TCR and CD8 molecules, which impair antigen recognition and lead to T cells anergy 62,63.

##### 3.3.1.4 Impairing immune cell trafficking and function

MDSCs express a disintegrin and metalloproteinase domain-containing protein 17 (ADAM17), which cleaves L-selectin (CD62L) from the surface of T cells. This restricts their capacity to home to lymph nodes and tumor sites. MDSC-derived NO can reduce E-selectin expression on blood vessels, which blocks T cell infiltration into tumors 64. Moreover, MDSCs can downregulate endothelial E-selectin and inactivate CCL2 chemokine to impede T-cell trafficking via NO and peroxynitrite 65,66. In addition to inhibit T cell activation and recruitment, MDSCs also suppress other immune cells to enable immunosuppression 67. They inhibit NK cell cytotoxicity through the above-mentioned factors including Arg1, ROS, NO and membrane-bound TGF-β 68. They impair DC maturation and antigen presentation, as well as dampening the development and antibody production of B cells. Moreover, MDSCs can promote immunosuppression by converting B cells into Bregs with enhanced T cell inhibitory function 69.

Taken together, MDSCs inhibit the function of T cells by expressing immunosuppressive molecules or shaping an immunosuppressive TME. Simultaneously, MDSCs can also suppress other immune cells to reinforce their immunosuppressive function.

#### 3.3.2 Non-immunological and pro-tumor functions of MDSCs

MDSCs not only contribute to tumor progression through immunosuppression but also via various non-immunogenic mechanisms, such as stimulating angiogenesis, inducing EMT, and facilitating the formation of pre-metastatic niches (Fig. 2).

##### 3.3.2.1 Promoting angiogenesis

A body of evidences indicate that MDSCs play a major role in the formation of tumor vascularization. They are a potent source of pro-angiogenic factors like VEGF and basic fibroblast growth factor (bFGF), which create a positive feedback loop that sustains their own accumulation and promotes angiogenesis 70. Furthermore, MDSCs can secrete several matrix metalloproteinases (MMPs), particularly MMP9, to facilitate endothelial cell migration via degrading the extracellular matrix to release sequestered VEGF 71. Also, studies show MDSCs produce other angiogenic mediators such as prokineticin 2 (PK2/Bv8) 72 and the chemokine CCL2 73. Notably, MDSCs have also been demonstrated to differentiate into endothelial-like cells and incorporate directly into the tumor vasculature 74.

##### 3.3.2.2 Inducing EMT and stemness

Accumulating evidences show that MDSCs actively improves the plasticity and aggressiveness of cancer cells by creating a stem-like state and EMT. They secrete a range of factors that activate critical signaling pathways in tumor cells. For example, MDSC-derived NO, IL-6, PGE2, and TGF-β can activate STAT3 and NOTCH signaling, which are crucial for inducing EMT program and maintaining cancer stemness 37. Intriguingly, the two MDSC subsets may have different spatiotemporal roles. M-MDSCs infiltrating the primary tumor is hypothesized to initiate EMT and tumor cells dissemination, whereas PMN-MDSCs at metastatic sites are thought to support the outgrowth of established metastases by enhancing cancer cell proliferation 75.

##### 3.3.2.3 Facilitating metastasis and pre-metastatic niche formation

Beyond the primary tumor, evidences show that MDSCs may contribute significantly to the metastatic cascade of tumor. They are reported to facilitate the initial dissemination of cancer cells by producing many proteolytic enzymes like MMP2, MMP9, and MMP13, which break down physical barriers for tumor invasion 76. While, in circulation, it is reported that PMN-MDSCs form clusters with circulating tumor cells (CTCs) to promote their dissemination and metastasis via activating ROS/Notch/Nodal signaling 77. Moreover, accumulating evidence suggests that MDSCs can be recruited to distant organs to form the "pre-metastatic niche", preparing the soil for future metastasis by series of mechanisms. One study indicates that MDSCs produce chemo-attractants, such as S100A8 and S100A9, to accelerate the spread of cancer cells from primary sites to secondary sites 78. Another study shows that MDSCs promote the adhesion of cancer cells on blood vessels and subsequent extravasation by increasing E-selection expression 79. What is more, MDSCs secret one key extracellular matrix-degrading enzyme MMP9 to support the incorporation of cancer cells into matrix of metastatic sites 80.

Overall, substantial data reveal that MDSCs accelerate tumor progression by series of immunosuppressive and non-immunological pro-tumor mechanisms (Fig. 2). For immunosuppressive functions, MDSCs increase immunomodulatory ligands and cytokines expression, manipulate the metabolic and chemical landscape of the TME and disrupt immune cell trafficking. While, as to the non-immunological pro-tumor roles of MDSCs, they encompass virtually all aspects of malignant progression, including promoting angiogenesis, enriching cancer stem cells, inducing EMT, and facilitating the formation of metastatic niches.

## 4. The involvement of MDSCs in RT

**Figure 3:**
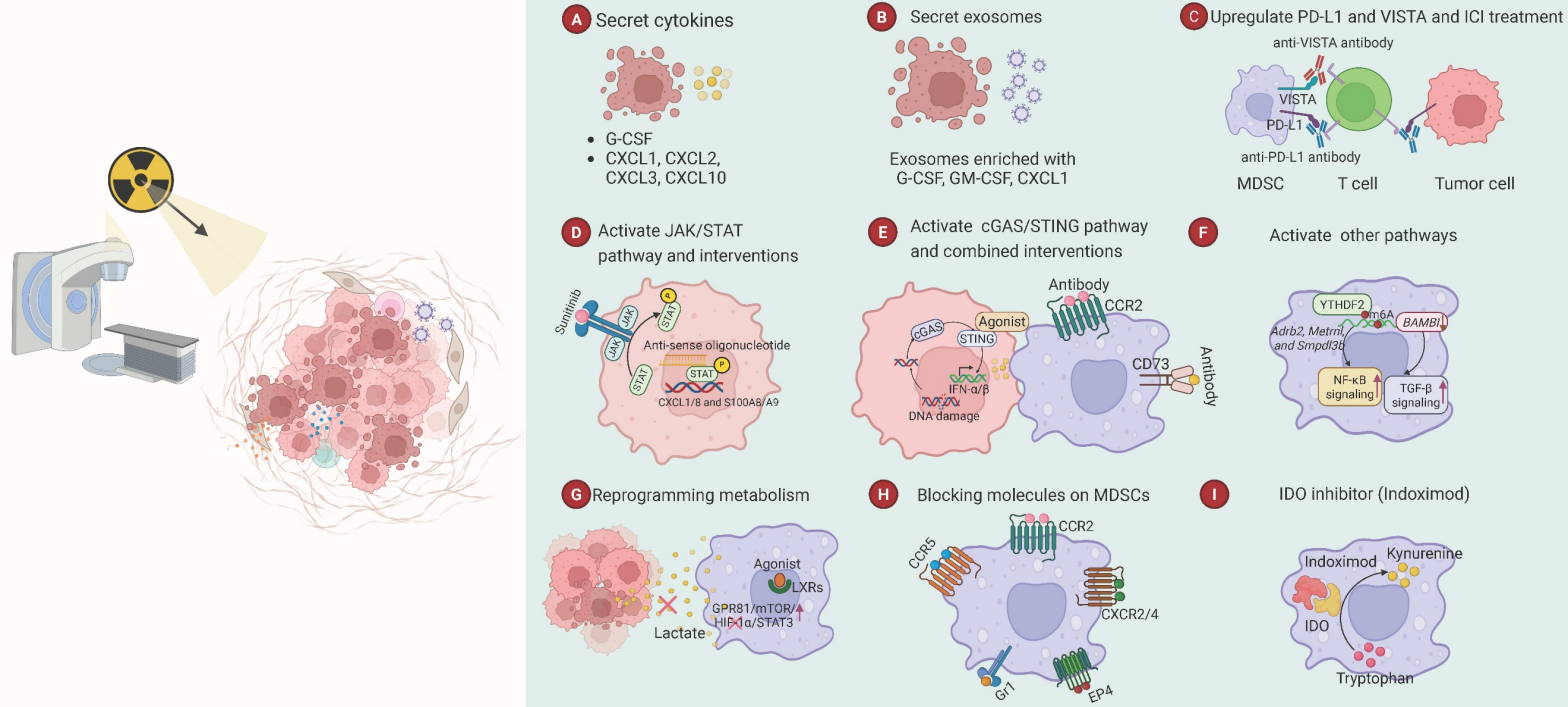
** The regulation of MDSCs by RT and MDSC-targeted strategies.** This figure illustrates the mechanisms of RT regulating MDSCs and representative strategies to target MDSCs for radiosensitization. (A) RT promotes the secretion of cytokines, such as G-CSF, CXCL1, CXCL2, CXCL3 and CXCL10. (B) RT promotes the release of exosomes enriched with cytokines, such as G-CSF, GM-CSF, and CXCL1. (C) RT upregulates the expression of PD-L1 and VISTA on MDSCs and other cells. Combing RT with ICI treatment improves therapeutic effect. (D) RT activates the JAK/STAT signaling pathway, which stimulates tumor cells to release cytokines to recruit and expand MDSCs. Inhibiting the pathway via STAT anti-sense oligonucleotides or the multi-kinase inhibitor Sunitinib can sensitize tumors to RT. (E) RT activates cGAS/STING pathway to promote anti-tumor immunity, but STING agonists alone are not enough to enhance RT. Additional blockade of MDSCs, such as the usage of anti-CCR2 or anti-CD73 antibodies, can enhance the treatment effect. (F) RT also activates other pathways, such as m^6^A, NF-κB, and TGF-β signaling pathways to boost the suppressive functions of MDSCs. The m^6^A reader protein YTHDF2 degrades mRNAs encoding NF-κB inhibitors, including Adrb2, Metrnl, and Smpdl3b, by m^6^A modification, thus activating the NF-κB pathway. YTHDF2 also degrades *BAMBI* mRNA by m^6^A modification, which activates the TGF-β pathway. (G) RT increases the lactate production of tumor cells. Lactate enhances the function of MDSCs by modulating the GPR81/mTOR/HIF-1α/STAT3 axis. Blocking lactate production or inhibiting HIF-1α suppresses MDSCs. Moreover, agonists of LXRs, the nuclear receptors involved in lipid metabolism, can inhibit the function of MDSCs and enhance the radiosensitivity of tumors. (H) MDSCs contain several kinds of surface receptors and molecules that mediate their migration and activation. Blocking these molecules, such as CCR2, CCR5, CXCR2/4, Gr1 and EP4, can improve the effectiveness of RT. (I) IDO metabolizes tryptophan into kynurenine, which enhances the immunosuppressive function of MDSC. The IDO inhibitor Indoximod has demonstrated potent potential to sensitize tumors to chemoRT in a Phase I clinical trial.

RT regulates MDSCs development, differentiation and recruitment. In reverse, MDSCs compromise the anti-tumor effects of RT. In this section, we overview how different RT regimens impact MDSCs. Importantly, we comprehensively summarize and classify the distinct mechanisms by which RT modulates MDSCs, comprising the involved cytokines and signaling molecules, common signaling pathways and metabolic reprogramming (Fig. 3). In addition, we also depict and summarize the differences of distinct subtypes of MDSCs involved in RT, and the synergy of MDSCs and other immunosuppressive cells involved in RT.

### 4.1 Regulation of different RT on MDSCs

Viewing RT as a universal driver of MDSC expansion is oversimplified. Recent evidence suggests this relationship is highly context- and modality-specific. As summarized by C. Jiménez *et al*., conventional fractionated RT (typically with doses <3 Gy per fraction) generally enhances MDSC expansion and recruitment 81, a trend corroborated by other studies 82-85. However, the immunological consequences of hypofractionated and other advanced radiotherapies are more complex and diverse (Table 3).

With the characteristics of larger doses per fraction and higher biological effectiveness, HypoRT is increasingly adopted because of the superior treatment efficiency 86,87. While, its effect on MDSCs is dualistic. In the models of triple-negative breast and colon cancers, HypoRT was found to facilitate the accumulation of MDSCs and reduce the infiltration of T-cells. Combining HypoRT with CXCR2 blockade to inhibit MDSCs migration greatly strengthened CAR-T cell efficacy 88. Moreover, when combined with anti-PD-L1 therapy, HypoRT but no single-dose radiotherapy conferred mice resistant to tumor rechallenge, which is partly mediated by Gr-1⁺ myeloid cells 16. Other studies show that high-dose RT could induce an early rise in MDSCs, but their depletion alone could not improve therapeutic outcomes 89. In contrast, another study revealed that the HypoRT reduced MDSCs in both blood and the tumor model of HCC via downregulating IL-6 and CCL5. The suppression of MDSCs potentially enhanced systemic anti-tumor immunity, inducing abscopal effects 90. To identify an optimal RT fractionation and dose to induce an immune response, different fractionation schemes were compared, during which the 8 Gy × 3 fraction was found to reduce splenic MDSCs and promote the infiltration of lymphocytes in tumor. This regimen, combining with anti-PD-1 therapy, effectively stimulates the abscopal effect 91. Ablative RT (ART), another high-dose regimen often delivered as SRS, SBRT, or stereotactic ablative body irradiation (SABR), enables precise delivery of doses as high as 30 Gy 92. One study indicates that a single 30 Gy dose promotes the infiltration of immunosuppressive cells such as MDSCs, TAMs, and Tregs 93. However, other data show that a single 30 Gy dose rapidly increased CD8⁺ T cell infiltration, eliminated stromal MDSCs, and thus induced tumor remission, which is not observed with daily fractionated irradiation 17. Likewise, SBRT was found to inhibit MDSC differentiation and trigger apoptosis via the miR-21/SORBS1 axis in a lung cancer model 94. In summary, the changes of MDSCs induced by photon RT is variable, and lack unified trends. However, comparisons within the same study, which utilize the consistent tumor models, offer more reliable insights. In HNSCC (head and neck squamous cell carcinoma) models, HypoRT appears more prone to increasing MDSCs than single-dose irradiation. Conversely, in colon cancer models, ablative RT seems more effectively deplete MDSCs than daily fractionated regimens.

Different types of rays have distinct effects on MDSCs. Compared to conventional photon RT, particle therapies, including proton and carbon ion beams, have superior physical precision to spare normal tissue and increase biological effectiveness against resistant tumors by taking advantage of the Bragg peak 95. One study demonstrated that proton radiation uniquely induces the infiltration of MDSCs into TME, whereas photon RT significantly upregulates PD-L1 expression. Consequently, combining photon RT with ICI conferred a significant survival benefit 96. Another study found that proton therapy increased mesothelin (MSLN) expression and thus significantly enhanced the infiltration of MSLN-targeting CAR-T in tumors. Meanwhile, combining RT and MSLN-targeting CAR-T remodeled the immunosuppressive TME by promoting antitumorigenic M1 macrophages polarization and reducing MDSCs 97. Carbon ion beams (5 GyE) were also shown to reduce MDSCs population and increase T cells, macrophages and NK cells, thereby stimulate anti-tumor immunity 98. BNCT is another special particle therapy, that selectively targets tumor cells through boron-loaded compounds and subsequent neutron irradiation to trigger a nuclear reaction. This process produces short-range, high-energy particles that effectively eliminate tumor cells and minimize damage to the surrounding healthy tissues, which provides a distinct advantage in the treatment of infiltrative and radioresistant cancers 26. A clinical case series demonstrated a strong positive correlation between circulating MDSCs level and tumor volume in patients with recurrent head and neck cancer, both before and after radiation therapy. These patients received BNCT and then fractionated IG-IMRT. The results indicate that the level of circulating M-MDSCs could be a marker for monitoring tumor progression in recurrent head and neck cancer patients following radiation therapy, including BNCT 99. Radiopharmaceutical therapy or targeted radionuclide therapy (TRT), has revealed benefits for treating metastatic cancer 100. A novel TRT, ^90^Y-NM600 was combined with androgen deprivation therapy (ADT) to treat metastatic prostate cancer and induced MDSCs accumulation significantly. The use of selective CXCR2 antagonist, reparixin, to suppress MDSCs recruitment, led to greater antitumor response 101. In summary, due to the lack of the parallel controls, we cannot drive the trend of particle RT and radionuclide modulating MDSCs. Therefore, further researches are needed to elucidate how these emerging RT modalities modulate immunity, especially their effects on key immunosuppressive cell populations such as MDSCs.

In short, the modulation of MDSCs by RT is not uniform, but is determined by radiation type, fractionation, dose, and tumor models. Data from limited parallel-controlled studies suggest that HypoRT may be more prone to increasing MDSCs than single-dose irradiation, whereas ablative RT appears more effectively depleting them compared to daily fractionation, though these observations warrant cautious interpretation. Notably, while the impact of RT on MDSCs is variable, this is particularly evident in unconventional regimens. In contrast, conventional photon RT with standard fractionation more consistently increases MDSCs within the TME (see parts below). Anyway, the complexity shows the need to strategically choose and combine specific RT modalities with complementary immunotherapies to counteract MDSC-mediated immunosuppression and activate systemic antitumor immunity.

### 4.2 The cytokines and signaling molecules involved in the regulation of MDSCs by RT

The differentiation, migration, and anti-tumor function of MDSCs are controlled by numerous cytokines and signaling molecules 102. RT similarly modulates MDSC activity by stimulating tumors to secrete various cytokines (Fig. 3).

#### 4.2.1 CSF, C-C and C-X-C motif chemokines and receptors

Tumor secret amount of CSF to recruit MDSCs, a process significantly augmented by RT (Fig. 3). For instance, RT upregulates the expression of G-CSF to recruit PMN-MDSC, which fosters radioresistance in tumors harboring mutant Nrf2^E79Q^ 103. Furthermore, the irradiated tumor cells can release exosomes enriched with G-CSF and GM-CSF, which may promote immunosuppressive premetastatic niches formation by recruiting M-MDSCs to the lung 83.

C-C and C-X-C motif chemokines and their receptors also are key mediators to recruit MDSCs to tumor sites or premetastatic niches (Fig. 3). RT can induce the secretion of various chemokines, such as CXCL1 83,85,101,103, CXCL2 101, CXCL3 103 and CXCL10 104, from tumors, which promote radioresistance and metastasis by recruiting MDSCs. Furthermore, RT triggers the release of exosomes enriched with CXCL1, which facilitates MDSC migration to the lung via the CXCL1/CXCR2 signaling, thereby fostering an immunosuppressive premetastatic niche 83. Consequently, therapeutic strategies that block these chemokine receptors, such as CXCR2 antagonists 101 or anti-CCR2 antibody 105, represent promising approaches to synergize with RT and improve patient survival.

#### 4.2.2 Immunosuppressive molecules

MDSCs can express immunosuppressive molecules, such as CTLA-4 and PD-L1 to dampen T cells activity 48,69,106,107 (Fig. 3). One study identified that the supernatant from irradiated head and neck squamous cell carcinoma cells upregulated the expression of PD-L1 on MDSCs. Furthermore, the combination of HypoRT but no single-dose RT with anti-PD-L1 treatment is resistant to tumor rechallenge by triggering ICD 16. However, the correlation between PD-1/PD-L1 expression and MDSCs accumulation post-RT seems context-dependent and varies according to treatment modality. For example, it's mentioned that photon radiation but not proton radiation significantly induced PD-L1 expression, whereas only proton radiation increased MDSCs population, suggesting distinct immune responses to proton therapy and photon RT 96. Moreover, local irradiation has been found to induce systemic expansion of MDSCs and increase PD-L1 expression on dendritic and myeloid cells, thereby promoting tumor progression and metastasis 104. Thus, ICI treatment sensitize RT is partly dependent on MDSCs.

### 4.3 Common signaling pathways in MDSCs regulation by RT

RT and other factors converge on common signaling pathways to regulate MDSCs. The shared pathways contain both canonical immune signaling pathways, such as NF-κB and cGAS/STING pathways, and crucial non-immunological pathways, especially those mediated by the STAT transcription factors family (Fig. 3).

The JAK/STAT signaling pathway, particularly with STAT3 as the core transcription factor, is pivotal for the development and function of MDSCs and also play a crucial role in RT 108. In cervical cancer, squamous cell carcinoma antigen1 (SCCA1, now referred to as SERPINB3) triggers STAT3 signaling to upregulate CXCL1/8 and S100A8/A9, thereby promoting MDSC infiltration and radioresistance 109. The use of carbon ion beams for melanoma-bearing mice was found to reduce the population of MDSCs in a JAK2/STAT3-dependent mechanism 98. Furthermore, targeting STAT3 using anti-sense oligonucleotide in combination with RT successfully prevented radiation-induced MDSCs recruitment, enhance effector T cell populations, and improve the treatment response in PDAC mouse model 110. Moreover, Sunitinib is a drug that decreases MDSC levels and sensitizes tumors to immunotherapy 111-113, which showed synergistic effects with SBRT in a clinical trial. This combination reversed the MDSC and Treg-mediated immunosuppression by reducing the levels of phosphorylated STAT3 in patients with oligometastases 114.

The cGAS/STING pathway is a pivotal innate immune defense mechanism in mammalian cells. It is initiated when the sensor cGAS detects aberrant cytosolic double-strand DNA, which derives from invading viruses or damaged host cells. Upon binding DNA, cGAS synthesizes a second messenger called 2'3'-cGAMP, which then activates the adaptor protein STING on the endoplasmic reticulum. Activated STING drives a downstream signaling cascade, which results in the generation of type I interferons and other inflammatory cytokines. This cascade is vital in the immune response to viral and tumor pathogens 115,116. In particular, local ablative radiation activates cGAS-STING pathway to enhance anti-tumor immunity by causing genomic DNA damage and subsequent cytoplasmic DNA leakage 117. However, RT-induced STING activation has been shown to promote resistance to local ablative radiation by recruiting MDSCs through the CCR2 pathway 105. Blocking CD73 simultaneously can counteract the immunosuppression and augment RT-induced innate immune response via the cGAS-STING pathway. As a consequence, the dual action creates a systematic CD8+ T cell-mediated antitumor immune response, and thus overcomes radioresistance 82. In addition, Zhang *et al*. developed an innovative bridging-lipid nanoparticle (B-LNP) that connects tumor-associated myeloid cells (TAMC) to glioblastoma cells via binding CD47 and PD-L1 simultaneously, promoting TAMC phagocytic activity. The powerful strategy reprograms TAMCs with enhanced phagocytic capacity by activating cGAS-STING pathway and stimulating a strong CD8+ T cell response, which effectively synergizes with RT 118.

Beyond the aforementioned pathways, RT also modulates MDSCs through other canonical signaling axes critical for tumorigenesis. NF-κB, TGF-β, and Wnt pathways, as well as epitranscriptomic regulation via N6-methyladenosine (m^6^A) modification, collectively promote inflammation, cell survival, and therapy resistance 119-122. An intriguing circuit involves NF-κB pathway and m^6^A modification. RT upregulates the m^6^A reader protein YTHDF2 via the NF-κB/RELA pathway. YTHDF2 then reinforces NF-κB activation by degrading transcripts of its negative regulators, such as *Adrb2*, *Metrnl*, and *Smpdl3b*. This IR-YTHDF2-NF-κB positive feedback loop augments the migratory and suppressive abilities of MDSCs, which contributes to radioresistance 123. Based on this finding, Zhang *et al*. demonstrated that YTHDF2 directly binds and degrades bone morphogenetic protein and activin membrane-bound inhibitor (*BAMBI*) transcripts in an m^6^A-dependent manner. Since BAMBI normally suppresses tumor infiltration and function of MDSCs via inhibiting TGF-β signaling, its downregulation augments MDSC-mediated immunosuppression. Notably, combining BAMBI depletion with IR not only improves local tumor control, but also suppresses distant metastasis, suggesting a systematic antitumor immunity 124. However, the role of TGF-β signaling is context-dependent and complicated. Contrary to the usual immunosuppressive function, a study revealed that TGF-β1 can modify MDSCs to lose suppressive function and develop a potent anti-tumor phenotype, which is characterized with improved antigen presentation and FAS-L-dependent cell death. Since RT can upregulate FAS expression on tumor cells, these modified TGF-β-MDSCs synergize RT by augmenting T-cell responses and result in tumor clearance 125. The methyltransferase RBM15 promotes radioresistance in non-small cell lung cancer (NSCLC) by creating an immunosuppressive microenvironment. RBM15 upregulates *CBR3-AS1* via an m6A-dependent mechanism, and this lncRNA then sponges miR-409-3p to trigger CXCL1-mediated MDSC recruitment and T-cell inhibition 85. Additionally, DKK1, a secreted inhibitor of Wnt signaling pathway, has been reported to foster radioresistance by facilitating the infiltration of MDSCs in head and neck squamous cell carcinoma 126.

Collectively, these common signaling pathways represent the potential targets to reverse MDSC-induced radioresistance.

### 4.4 RT modulating MDSCs by reprogramming metabolism

RT further regulates MDSCs by altering the metabolic landscape of the TME (Fig. 3). Depleting L-arginine by Arg1 in the TME is a primary mechanism of MDSCs to suppress T cells. One study has demonstrated that irradiation recruits PMN-MDSCs and upregulates Arg1 expression, thus suppressing CD8^+^ T cell response. Notably, phosphodiesterase 5 (PDE5) inhibitor sildenafil reverses these effects, reducing both MDSCs accumulation and Arg1 expression, and consequently abrogating MDSCs-mediated immunosuppression 127. Moreover, RT enhances tumor lactate secretion, which activates MDSCs through the GPR81/mTOR/HIF-1α/STAT3 signaling axis. Importantly, inhibiting lactate production in tumor cells or depleting Hif-1α in MDSCs boost antitumor T-cell responses and efficiently inhibited tumor progression after RT 128. Intriguingly, it's reported that malnutrition is correlated with an increased proportion of MDSCs in the peripheral circulation of patients with head and neck squamous cell carcinoma, which may contribute to RT failure 129. Summarily, RT can reprogram metabolism within the TME, which regulates MDSC accumulation and function, and ultimately influences RT outcomes. Reversing these metabolic alterations represents a promising strategy to overcome radioresistance. Furthermore, future works are needed to investigate whether RT modulates MDSCs via other metabolic pathways, including enhancing the production of immunosuppressive adenosine and ROS/RNS.

### 4.5 Different subtypes of MDSCs involved in RT

As aforementioned, MDSCs can be predominantly classified into two subtypes, PMN-MDSCs and M-MDSCs. Since PMN-MDSCs and M-MDSCs exert distinct function in tumor progression, it is valuable to delve into how RT impacting two distinct subtypes of MDSCs.

Generally, lines of evidences show that RT seems to impact distinctly on PMN-MDSCs and M-MDSCs in different conditions. Some studies found that RT is prone to recruit PMN-MDSCs into TME 103,127,130. PMN-MDSCs repress the CD8+ T cells response by elevating Arg1 expression 127. Additionally, CCR5 antagonist maraviroc can potently abrogate PMN-MDSC-mediated immunosuppression 130. NFE2L2 mutations enhance radioresistance in head and neck cancer by increasing recruitment of intratumoral PMN-MDSCs and decreasing M1-polarized macrophages 103. However, the role of M-MDSCs during RT seems paradoxical, though more evidences suggest RT increase M-MDSCs, which leads to tumor progression. Radiation-induced STING activation is immunosuppressive due to M-MDSC infiltration, leading to radioresistance 105. A case series highlights that circulating M-MDSC level is positively associated with tumor size before BNCT and one month after the last IG-IMRT treatment in recurrent head and neck cancer patients 99. Unexpectedly, RT markedly enhanced M-MDSCs recruitment but not PMN-MDSCs to the lung in mice with breast cancer, resulting in pre-metastasis formation 83. However, locally advanced rectal cancer (LARC) patients resistant to RT display an obvious decrease in M-MDSC and an increase of Tregs after short-course preoperative radiotherapy (SC-RT) 131.

In summary, RT exert complex and sometimes paradoxical effects on the recruitment and function of distinct MDSC subtypes, varying on cancer types, radiation regimen, and specific TME possibly. More studies are needed to illustrate how RT influence different MDSC subtypes and whether intervention methods should be separate or unified.

### 4.6 The synergy of MDSCs and other immunosuppressive cells involved in RT

Immunosuppressive populations, primarily MDSCs, Tregs and TAMs, typically accumulate concurrently and engage in a self-reinforcing crosstalk. These immunosuppressive cells engage in dynamic crosstalk, mutually sustaining their survival and expansion, which synergistically drive tumor progression. For instance, MDSCs secrete immunosuppressive cytokines, such as TGFβand IL-10, to facilitate Tregs induction. In turn, Treg-derived TGF-βand IL-10 sustain and reinforce MDSC-mediated immunosuppression 132. In addition, both MDSCs and TAMs are recruited to the TME by tumor-derived cytokines, and they also secret numerous soluble cytokines, such as CSF, IL-10, CCL2, CCL5, TGF-βand IL-6 to support each other's survival and expansion. Notably, M-MDSCs can further differentiate into TAMs, obtaining a more stable immunosuppressive phenotype 133. In summary, these immunosuppressive cells collaborate to establish a self-reinforcing immunosuppressive network that ultimately exerts potent pro-tumor activity.

Several lines of evidences demonstrate that RT promotes the accumulation not only of MDSCs but also of other immunosuppressive cells, including Tregs and TAMs 89,109,131. Notably, the concomitant enrichment of these cells may limit the therapeutic efficacy of combining RT with MDSC-targeted approaches. For instance, when synergizing with SBRT, the effects of sunitinib on reducing the accumulation and immune-suppressive function of MDSCs are correlated with Treg reduction, in responders but not in nonresponding patients 114. In prostate cancer models, systemic MDSCs depletion failed to augment the antitumor efficacy of high-dose radiotherapy due to a compensatory Treg response 89. In contrast, some strategies that simultaneously target MDSCs, Tregs or TAMs have obviously augmented RT outcomes. These methods include CD73 blockade 82, nanoformulated TLR 7/8 agonist and PI3K delta inhibitor 134, fibroblast activation protein-alpha (FAPα)-based cancer vaccine 135, diABZI-loaded B-LNPs 118, BL@SeNPs 136, among others. Taken together, RT-induced accumulation of immunosuppressive cells extends beyond MDSCs, often involving Tregs and TAMs as well. Therefore, therapeutic strategies that singularly target MDSCs may be insufficient to fully potentiate RT. A more promising approach lies in "cocktail" regimens, which are designed to concurrently inhibit multiple immunosuppressive cell types, including MDSCs, Tregs, and TAMs.

To sum up, RT exerts complex and context-dependent effects on MDSCs, varying with radiation regimens and tumor types. Mechanistically, RT modulates MDSCs by stimulating cytokines secretion, activating common signaling pathways like NF-κB, cGAS/STING, and STAT, and reprogramming metabolic processes within the TME. These interconnected mechanisms jointly influence MDSCs accumulation and functions, highlighting the need for combining targeted strategies with RT to reverse their pro-tumor effects. Furthermore, since RT-induced immunosuppression extends beyond MDSCs to include Tregs and TAMs, future therapeutic strategies should adopt a "cocktail" approach to simultaneously inhibit multiple immunosuppressive cell populations.

## 5. Therapeutics targeting MDSCs to synergize RT

The pathological accumulation and activation of MDSCs before and after RT significantly compromise its anti-tumor efficacy. This not only helps tumor radioresistance but also promotes metastatic dissemination. In order to synergize RT, a substantial number of therapeutic strategies have been developed to target MDSCs. Based on the mechanisms by which RT modulates MDSCs development and recruitment, these strategies can be broadly categorized as follows (Fig. 3) (Table 4).

### 5.1 Blocking the chemokine receptors

Blocking chemokine receptors remains an effective strategy for inhibiting MDSCs. For instance, the CCR5 antagonist maraviroc has been shown to inhibit the differentiation of bone marrow cells into PMN-MDSCs, attenuate their migration and suppression of T-cell proliferation. When combined with conventional fractionated RT (CFRT), maraviroc potently inhibited hepatocellular carcinoma growth in tumor-bearing mice, via MDSC suppression probably 130. When added to a combination of ADT and TRT, the CXCR2 antagonist reparixin was reported to improve anti-tumor effect by inhibiting MDSC recruitment 101. Subsequent analysis has shown that CXCR2 inhibition, in conjunction with HypoRT, markedly reduced MDSCs migration into tumors and substantially improved the intratumoral infiltration and therapeutic effectiveness of CAR-T cells 88. Anlotinib is a multi-target tyrosine kinase inhibitor that acts on vascular endothelial growth factor receptor (VEGFR), platelet-derived growth factor receptor (PDGFR), fibroblast growth factor receptor (FGFR), and stem cell growth factor receptor (C-KIT) kinases. It modifies the TME when used in conjunction with RT and a PD-1 inhibitor by diminishing PMN-MDSCs and augmenting T cell populations, thereby producing significant anti-tumor effects 137. Prostaglandin E2 (PGE2) promotes immune suppression by activating its receptors, skewing myeloid cell differentiation toward MDSCs rather than DCs 138. The highly potent prostaglandin E receptor (EP4) antagonist ASP7657 increases intratumoral DC and CD8+ T-cell populations while decreasing M-MDSCs, thereby enhancing antitumor immunity in combination with RT or anti-PD-1 therapy 139. One study revealed that although cGAMP, a product of cGAS and a STING agonist, can synergize with IR to trigger a robust anti-tumor response, tumor relapse occurred in some models. This relapse was caused by an influx of CCR2^+^Ly6C⁺ M-MDSCs, which was mediated by interferon-induced immunosuppression. Treatment with anti-CCR2 antibody improved this immunosuppression after STING pathway activation, which improved the combined anti-tumor efficacy of STING agonist and RT 105. Targeting surface receptors on MDSCs is a promising therapeutic strategy. However, it must be taken into consideration that blocking a single receptor may have a limited impact on the function of MDSCs. Moreover, these receptors are not limited to MDSCs. For instance, CCR2, one of the most extensively studied receptors on MDSCs, is also expressed on other immune cells 140. Thus, the side effects of these agents on normal immunity should be carefully assessed.

### 5.2 Blocking the surface molecules on MDSCs

Gr1 is an epitope that exists both in Ly6G and Ly6C proteins. PMN-MDSC is characterized with Ly6G^+^ Ly6C^low^, while the M-MDSC has a feature of Ly6G^-^Ly6C^high^ 41. Blocking Gr-1 with a specific antibody successfully depletes MDSCs in murine prostate tumor models, which leads to enhanced antitumor response following ADT synthesized with TRT 101. Similarly, anti-Gr1 antibody synergizes with RT to induce abscopal effects in mouse bladder cancer models 141. However, since Ly6G and Ly6C are not exclusive markers for MDSCs over normal granulocytes and monocytes, so using Gr-1 antibody might accidentally impair normal immune function.

### 5.3 ICIs and immune agonists

To synergize RT, ICIs and immune agonists also hold significant therapeutic potential. Preclinical evidences show that combining RT with ICIs shows significant synergistic effect, partly due to effects on MDSCs. However, the interaction is complex and modality-dependent. For instance, while proton RT induces MDSCs accumulation and photon RT upregulates PD-L1 expression, a significant survival benefit was only achieved by combining anti-PD-L1 with photon RT, not with proton RT 96. However, another study demonstrates the combination of proton therapy and anti-PD-L1 treatment also obviously delays the growth of mice hepatocellular carcinoma. While, evidences indicate that proton therapy upregulates PD-L1 and recruits MDSCs more significantly than photon RT 142. Additionally, local irradiation increases systemic MDSCs level and elevates PD-L1 expression on dendritic and myeloid cells by CXCL10 signaling. Blocking either the PD-L1/CXCL10 axis or MDSCs infiltration during irradiation augments abscopal tumor control and reduces metastasis 104. VISTA is another crucial immune checkpoint molecule that expresses on several kinds of immune cells, which contributes to immune evasion in cancer by inhibiting T cell function 143. RT increases VISTA expression on MDSCs 84,144,145, and its high levels correlate with poor prognosis in cancers, such as NSCLC 145. RT combined with anti-VISTA antibodies repolarizes immunosuppressive myeloid cells and enhances intratumoral T-cell function, which reveals synergistic efficacy in preclinical models of head and neck, breast, and colorectal cancers 84. This synergy is reinforced by the finding that RT significantly increases VISTA expression on TANs, and combining VISTA blockade with RT markedly decreases overall immunosuppressive myeloid populations 145. Additionally, focal RT was demonstrated to reinforce the effect of a VISTA-specific antibody in mice triple-negative breast cancer (TNBC) 144. ICI therapy reverses the immunosuppressive TME and thus reinforces the effect of both chemotherapy and RT. However, the clinical practice seems more complicated than laboratory data. The unsatisfactory results might owe to the fact that the upregulation of PD-1/PD-L1 is just one of the roles that RT takes in tumor immunosuppression. Beyond the ICIs, immune agonists such as interleukin-12 (IL-12) actively stimulate the anti-tumor immune response to cooperate with RT 146. In murine hepatocellular carcinoma, combining RT and IL-12 treatment more significantly suppresses tumor growth compared to any monotherapy. Mechanistically, the combination of RT and IL-12 promotes DCs to present antigens and diminishes the suppressive capacity of MDSC by eliminating ROS production 147. Since the expression of immunosuppressive molecules is the core mechanism by which MDSCs suppress antitumor immunity, ICIs represents a promising strategy to sensitize tumors to RT. However, considering the unsatisfactory clinical outcomes of combining RT with ICIs thus far, integrated strategies reversing RT-induced immunosuppression should be further considered.

### 5.4 Restoring metabolic homeostasis

RT regulates MDSCs through metabolic reprogramming, and MDSCs, in turn, utilize the metabolic alterations to promote tumor progression. Targeting the metabolic vulnerabilities of MDSCs is a prospective approach to restore immune homeostasis and augment the effects of RT. One crucial metabolic signaling cascade is the adenosine pathway. Teaming up with CD39, CD73 catalyzes adenosine monophosphate (AMP) to produce immunosuppressive adenosine, which can immensely suppress immune responses 148. It has been revealed that RT upregulated CD73 expression by the ataxia telangiectasia and Rad3-related (ATR) pathway. The integration of RT with CD73 blockade effectively reverses the immunosuppressive TME and invigorates CD8^+^ T cell-driven, specific antitumor immune responses 82. Since the accumulation of adenosine in the TME potently suppress antitumor immune response, targeting adenosine metabolic enzymes represents a promising strategy to sensitize tumor cells to RT. Fatty acid metabolism is also crucial for MDSCs development and function. Polyunsaturated fatty acids promote the expansion of MDSCs by activating the JAK/STAT3 pathway 149. The fatty acid transport protein 2 (FATP2) regulates the production of ROS in MDSCs and confers them the immunosuppressive function 62. Liver-X nuclear receptors (LXRs), including LXRα and LXRβ, are members of the oxysterol-activated transcription factors, which promote the transcription of ApoE and other genes involved in lipid metabolism 150. Therapeutic LXRs agonists have been reported to reduce MDSCs abundance in murine tumor models and in patients 151. Furthermore, combining the LXRs agonists, GW3965 and RGX-104, significantly sensitizes the RT in NSCLC via promoting MDSCs apoptosis and activating cytotoxic T lymphocyte (CTL) and T-helper 1 (Th1) in TME 152. Metabolic reprogramming is a hallmark of malignant tumor progression, and targeting metabolism has long been a central focus in cancer therapeutic research. For example, metabolic agents like arginase inhibitor INCB0158 and CD73 antibody oleclumab are being evaluated in clinical trials with ICI therapy, showing promising antitumor potential 153,154. Therefore, investigating the functional impact of metabolic intervention of MDSCs and its potential to enhance radiosensitization is a promising and valuable research direction.

### 5.5 Nanoparticles-based strategies

Nanoparticles provide a flexible platform to precisely target MDSCs and the immunosuppressive TME, thus improving the effectiveness of RT. Their design allows for the simultaneous delivery of agents targeting multiple pathways. For example, polymeric micellar nanoparticles (PMNPs) co-encapsulating Toll-like receptor 7/8 (TLR7/8) agonist and phosphoinositide 3-kinase delta (PI3Kδ) inhibitor greatly improve the anti-tumor effect of RT by promoting M1 macrophage polarization and inhibiting MDSCs accumulation 134. Besides drug delivery, nanoparticles can directly improve key immunosuppressive mechanisms. A pH-sensitive catalase-gold nanoaggregate (Au@CAT) was developed to collect ROS during RT. This combination with low-dose RT effectively reduced infiltrating MDSCs, M2 macrophages, and Tregs, improving anti-tumor outcomes 155. Innovative designs also allow for complex cell engagement. A bridging-lipid nanoparticle (B-LNP) was developed to concurrently target tumor-associated myeloid cells and glioblastoma cells via anti-CD47/PD-L1 binding. When loaded with STING agonist diABZI, these B-LNPs synergize with RT to induce brain tumor regression and establish anti-glioma immunological memory in preclinical models 118. Furthermore, a cationic polymer-modified magnetic nanoparticle platform has been shown to synergize with RT by destroying glioma cells and repolarizing immunosuppressive phenotypes, with uptake by MDSCs in the brain tumor confirmed via its magnetic properties 156. However, the precise mechanism by which this magnetic platform reprograms MDSCs to attack tumor cells remains unclear 156. Given that MDSCs are a heterogeneous cell population that exert immunosuppression via multiple mechanisms, a feature that's fundamentally different from targeting a single molecule or pathway. Furthermore, considering their capacity for multi-drug delivery, multifunctional nanocarriers are promising tools for comprehensive MDSCs suppression.

### 5.6 Other MDSC-targeted strategies to enhance RT efficacy

Beyond the previous discussed approaches, some other strategies are being considered to improve RT efficacy. Cancer vaccines aimed at altering the immunosuppressive TME can work with RT. For example, combining a fibroblast activation protein-alpha (FAPα)-targeted vaccine with SABR recruits and activates CD8⁺ T cells against both tumor and stromal cells, reprograms macrophages to M1 phenotype, and depletes MDSCs and Tregs, resulting in significant tumor growth suppression and prevention of metastasis 135. Likewise, chimeric antibody bavituximab has anti-angiogenic capacity and can also modulate immune system 157. A phase 2 clinical trial in glioblastoma (GBM) first demonstrates bavituximab decreases intratumoral MDSCs. Moreover, high level of pre-treatment myeloid transcript correlates with improved progression-free survival (PFS) and overall survival (OS) when bavituximab is combined with RT 158. Small molecule inhibitors represent another avenue for targeting MDSCs-associated pathways. The glutaminase inhibitor CB839 can reverse Nrf2^E79Q^-induced cytokine changes post-RT by regulating TLR4 expression, which results in decreased MDSC recruitment and augments RT response 103. Tadalafil is an FDA-approved phosphodiesterase 5 (PDE5) inhibitor, which has been revealed to reverse radiation-induced MDSCs accumulation and improved survival in several preclinical GBM models 159-161. However, a phase Ib trial involved the combination of Tadalafil and RT did not show a significant difference in PFS or OS compared to historical controls in GBM patients 162. Emerging agents also show potential. A secondary mitochondrial-derived activator of caspase (SMAC) mimetic Debio 1143 was reported to augment the effect of ablative RT by triggering a tumor-specific Tc1/cytokine response and eliminating immunosuppressive myeloid cells from the TME 93. Though evidences from these studies suggest that alterations in MDSCs are associated with synergistic responses to RT, the specific pathways through which these agents modulate the pro-tumor functions of MDSC require further investigation.

Taken together, various strategies aimed at MDSCs suppression have been developed to enhance RT efficacy and have demonstrated translational potential (Fig. 3) (Table 4). However, the limitations of these preclinical studies should be taken into account. A major concern is the risk of off-target effects, as surface receptors, such as CCR2 and Gr-1, express both on MDSCs and normal immune cells. Moreover, though some strategies targeting MDSCs display encouraging, clinical translation has proven difficult so far, such as the combination of ICIs with RT. The disconnect between laboratory findings and clinical efficacy indicates the limitations of current preclinical models, which often fail to fully recapitulate the complex TME in humans. Notably, since RT-induced immunosuppression through multiple cell populations, mainly including MDSCs, Tregs and TAMs, a "cocktail" approach should be adopted to simultaneously inhibit these immunosuppressive subsets.

### 5.7 Clinical trials of combining RT and MDSCs-targeted strategies

As outlined above, numerous studies have elucidated the mechanisms that RT modulates MDSCs and MDSCs contribute to RT resistance. To translate the preclinical outcomes into clinical practice, a number of phase I/II clinical trials involving the combination of RT and MDSC-targeting interventions have been initiated (Table 5 and Table 6).

However, it is important to note that the complexity of immune system and the lack of specific MDSC markers compromise the specificity of these approaches. Furthermore, we eliminate some related clinical trials without the published results from our tables, which may introduce a selection bias into our analysis. ICI therapies, such as antibodies targeting PD-1 and PD-L1, can significantly reverse the immunosuppressive function of MDSCs to sensitize RT in preclinical models 96,104,142, while the clinical trials have mixed results 163. Actually, the clinical outcomes of combining RT with ICIs have fallen short of expectations 163. It is worthy to take the strategies into account that eliminating other immunosuppressive cells, such as MDSCs, Tregs, TAMs, and TANs, which may achieve substantial therapeutic synergy between RT and other therapies.

Besides ICI-based strategies, current clinical trials are exploring a lot of approaches to clear or inhibit MDSCs, like targeting CXCR4, CD73, COX2, IDO/IDO1, and Retinoic Acid Receptor (Table 4). Overall, the side effects of these MDSC-targeting interventions with RT are usually manageable. But preliminary observations show that most of these agents haven't given many clinical benefits, and only a bit of improvement. For instance, although the COX2/PGE2 signaling pathway mediates the immunosuppressive functions of TAMs and MDSCs 164 and elicits a robust T-cell response in melanoma-bearing mice when combined with DC vaccination 165, the COX-2 inhibitor Celecoxib did not improve OS or PFS in rigorously controlled clinical trials 166. Similarly, in trials combining Celecoxib with RT, the observed benefits appear minimal 167-170. The chemokine CXCL12 recruits MDSCs into tumor tissues by binding to their highly expressed receptor CXCR4 171. In two single-arm clinical trials involving patients with malignant brain tumors, the combination of the CXCR4 antagonist Plerixafor with chemoRT showed no significant clinical benefits compared to historical controls 172 (NCT03746080, NCT01977677). IDO not only recruits MDSCs by metabolizing tryptophan into kynurenine, but also mediates MDSC immunosuppression by inhibiting T-cell function and promoting Treg formation 57,173. However, in a critical Phase III trial, the IDO1 inhibitor Epacadostat didn't improve the response rate of pembrolizumab in patients with unresectable or metastatic melanoma 174. When combined with RT, Epacadostat also didn't increase the abscopal response rate (NCT03322384) or provide survival benefits (NCT03532295). Notably, the IDO pathway inhibitor Indoximod showed promising preliminary efficacy in pediatric brain tumors 175.

All-trans retinoic acid (ATRA) activates the RAR, driving the differentiation of MDSCs into mature, normally functional myeloid cells, thus disrupting their immunosuppressive functions 176. Several clinical trials have confirmed that ATRA significantly reduces MDSC levels and enhances antitumor immunity 177-179. Although less toxic, combining 13-cis-RA with IFNα and RT failed to provide a survival advantage over standard chemoradiation in a phase II cervical cancer trial 180. An ongoing Phase III trial combing ATRA with RT may offer further insights into therapeutic value of this approach, and it is the only advanced clinical investigation involving the combination of RT and MDSCs-targeting (NCT06706401).

Other strategies that might affect MDSC numbers and function, like blocking the survival receptor CSF-1R 181, inhibiting the adenosine-producing enzyme CD73 182, and neutralizing the MDSC chemokine IL-8 183, are being actively investigated for their safety and potential to improve the clinical outcomes of RT and other treatments (Table 6).

To date, all clinical trials combining MDSC-targeted therapies and RT are in early phase, and most of them have limited clinical value. But, optimizing trial designs, like the timing of combination with RT, RT modalities, and the integration of ICIs or inhibitors of other immunosuppressive cells, could be a promising direction for further exploration.

In summary, various MDSCs-targeted strategies have been developed to sensitize RT, which include blocking chemokine receptors and other surface molecules, adopting immune checkpoint inhibitors, restoring metabolic homeostasis, and utilizing nanoparticles-based strategies, as well as other MDSC-targeted approaches. Though pre-clinical data display great potential to combine these strategies with RT, clinical trials have shown limited efficacy.

## 6. Conclusion and Perspectives

As detailed in this review, the interplay between RT and MDSCs is intricate and reciprocal. Substantial evidences confirm that RT promotes the expansion, recruitment and activation of MDSCs, which in turn contributes to radioresistance, limits the incidence of abscopal effects, and even facilitate tumor metastasis. Therefore, diverse preclinical strategies targeting MDSCs have been investigated to synergize with RT. They include blocking chemokine receptors and surface molecules, combining with ICIs and immune agonists, restoring metabolic homeostasis, and applying new methods such as nanoparticles and cancer vaccines. In preclinical tumor models, these strategies have demonstrated promising potential in augmenting RT efficacy.

However, the translation of these findings into clinical benefit has proven challenging. To date, the agents harnessed to target MDSCs generally lack specificity, and inhibiting MDSCs is just a secondary consequence of their primary therapeutic intent. Moreover, the precise mechanisms by which these agents suppress MDSCs remain insufficiently elucidated. While generally tolerable, their clinical efficacy in conjunction with RT has been modest. However, it would be premature to conclude that targeting MDSCs cannot further augment the effects of RT.

Several fundamental challenges complicate the investigation of MDSCs and their relationship with RT. First, due to the inherent plasticity and heterogeneity of MDSCs, it is pretty difficult to analyze their characteristics and function. Currently, no phenotypic markers can reliably distinguish MDSCs from classical neutrophils or monocytes. Therefore, identifying MDSCs mainly depends on their immunosuppressive ability, particularly the inhibition of T cell function 40. Second, the impact of RT on MDSCs is context-dependent, influenced by radiation modality, dose, fractionation, tumor type and the TME. Evidences also show that the level and function of MDSCs differ at distinct time points after irradiation 81. Third, there is significant differences between murine and human immune systems. However, preclinical studies investigating the relationship of RT and MDSCs have not utilized humanized immune system models. This fundamental limitation may, in part, account for the disappointing outcomes observed in clinical trials. Fourth, current standard-of-care regimens of tumors frequently involve multiple combinations, such as chemotherapy, RT, and immunotherapy, rather than RT alone. However, preclinical studies usually simplify the combination of RT and MDSCs-targeted strategies, and the effect cannot be reproduced in complicated human patient settings. Moreover, most clinical trials to date have not rigorously evaluated the specific effects of the tested interventions on MDSC function, so it's hard to link any clinical outcome directly to MDSC modulation. Finally, RT-induced accumulation of immunosuppressive cells extends beyond MDSCs, often involving Tregs and TAMs as well. Therefore, therapeutic strategies targeting MDSCs in isolation may be insufficient to fully overcome RT-induced immune suppression. In view of these challenges, definitive conclusions regarding the clinical value of MDSCs-targeted strategies to overcome radioresistance remain elusive. Uncovering the characteristics and roles of MDSCs in radioresistance and tumor progression is still on the way.

Future research must continue to prioritize robust preclinical validation. High-resolution platform, such as single-cell sequencing technology and spatial transcriptomics, offers a more effective platform to analyze the heterogeneity and variability of the MDSC populations. Identifying specific surface markers of MDSCs is key to exploring the functions of MDSCs. Based on this, further researches are need to compare the effects of different RT modalities on MDSCs and the dynamic changes of MDSCs at various time points post-irradiation. Meanwhile, using *in situ* animal models and standard treatment regimens will inform the subsequent clinical researches more accurately. Developing targeted intervention strategies to suppress MDSCs selectively is the main concern, which holds theoretical potential to enhance therapeutic efficacy while minimizing off-target effects. Finally, the characteristics and function of MDSC should be further revealed in the context of new technologies, and their clinical values under the standard treatment paradigm also need to be further explored.

## Figures and Tables

**Table 1:** Summary of RT types and characteristics

Classification dimension	Type	Key characteristics	Main advantages	References
Technical principle and precision	Conventional RT	Traditional technology, lower precision, simple field shapes.	Widely available, lower cost.	1
	3D Conformal RT (3D-CRT)	Uses CT/MRI for 3D planning to conform dose to the target volume.	Better normal tissue sparing than conventional RT.	1
	Intensity-Modulated RT (IMRT)	Modulates beam intensity across each field.	High precise dose distribution.	18
	Volumetric modulated arc therapy (VMAT)	Modulates beam intensity across multiple static fields/angles directed at the tumor.	High precise and conformal dose distribution.	19
	Image-guided RT (IGRT)	Incorporates imaging before or during treatment to verify and correct target position.	Managing inter-fraction motion and set-up errors, enabling high-precision delivery.	20
	Stereotactic RT (SRS/SBRT)	Precise, accurate, ablative. Few fractions, high dose per fraction, highly concentrated dose with a sharp dose gradient.	Short treatment course, potent biological effect, ablative potential.	21
	Particle therapy (proton radiation/ heavy ion particle therapy)	Uses particles (protons, ions). Exhibiting the "Bragg Peak" physical effect.	Minimal to no exit dose, maximizing protection of normal tissues beyond the target.	22
Dose fractionation	Conventional fractionation	1.8-2.0 Gray (Gy) per fraction, once daily, 5 fractions per week.	Classic model, allows normal tissue repair and tumor re-oxygenation.	1
	Hypofractionation	Higher dose per fraction, resulting in a lower total number of fractions.	Shorter overall treatment time; potentially enhanced biologic effect on certain tumors.	1
	Hyperfractionation / Accelerated RT	Smaller dose per fraction but more than 1 fraction per day; with or without reduced overall treatment time.	Aims to overcome tumor re-population.	1
Delivery method	External beam RT (EBRT)	Radiation source is located at a distance from the body.	Versatile, covering a wide range of techniques and targets.	1
	Brachytherapy (Internal)	Radioactive sources are placed inside or very close to the tumor.	Very high, localized dose to the target with rapid dose fall-off to spare surroundings.	23
	Radionuclide therapy	Systemic administration of radioactive drugs that target tumors.	Systemic targeted therapy for disseminated disease.	24
Emerging modality	FLASH RT	Ultra-high dose rate irradiation (>40 Gy/s). Delivers the entire dose in a fraction of a second. Induces the "FLASH effect".	Achieves equivalent tumor kill compared to conventional RT while dramatically reducing damage to healthy tissues.	25
	Boron neutron capture therapy (BNCT)	A boron drug collects in the tumor, which is then irradiated with neutrons to trigger a cell-killing nuclear reaction.	Destroys cancer cells with microscopic precision while sparing healthy tissue.	26

**Table 2:** Minimal phenotypic characteristics necessary to identify MDSCs

Characteristic	Neutrophils	Monocytes	PMN-MDSCs	M-MDSCs
Standard phenotypical markers in mice	CD11b^+^Ly6G^+^Ly6C^lo^	CD11b^+^Ly6G^-^Ly6C^hi^	CD11b^+^Ly6G^+^Ly6C^lo^	CD11b^+^Ly6G^-^Ly6C^hi^
Novel markers in mice	NA	NA	CD11b^+^Ly6G^+^CD84^+^	CD11b^+^ Ly6G^-^Ly6C^hi^CD84^+^
Standard phenotypical markers in humans	CD11b^+^CD14^-^CD15^+^ /CD66b^+^; High-density cells	CD14^+^CD15^-^HLA-DR^hi^	CD11b^+^CD14^-^CD15^+^/ CD66b^+^; Low-density cells	CD14^+^CD15^-^HLA-DR^lo/-^
Novel markers in human	NA	NA	CD15^+^/CD66b^+^CD14^-^ LOX1^+^; CD15^+^/CD66b^+^ CD14^-^CD84^+^	CD14^+^/CD66b^-^CXCR1^+^ ; CD14^+^/CD66b^-^CD84^+^

NA, not applicable.

**Table 3:** Effects of different RT modalities on the expansion and recruitment of MDSCs

Energy Modality	Type/Name	Fractionation and Dose	Impact on MDSCs/PD-L1	Combination Strategy	Mouse Model	Reference
Photon RT	Hypofractionated RT	5 Gy × 5F	Increasing MDSCs	Combining HypoRT, CXCR2 blockade and CAR-T	TNBC and colon cancer	88
	Hypofractionated RT	9 Gy × 3F	Increasing MDSCs and PD-L1	Combining HypoRT and anti-PD-L1 treatment	HNSCC	16
	Single-dose RT	20 Gy×1F	Don't changing MDSCs	-	HNSCC	16
	High-dose RT	8 Gy × 1/2 F	Inducing an early rise of MDSCs and upregulating Tregs	Dual targeting MDSCs and Tregs to sensitize RT	Prostate cancer	89
	Hypofractionated RT	8 Gy × 3 F	Reducing splenic MDSCs	Combining Hypofractionated RT, MDSCs depletion and anti-PD-1 therapy	Colon cancer	91
	Hypofractionated RT	Higher than 4 Gy × 10 F	Reducing MDSCs in blood and tumor	Hypofractionated RT results in abscopal effects	HCC	90
	Ablative RT	30 Gy × 1 F	Increasing MDSCs	Combining a secondary mitochondrial-derived activators of caspase (SMAC) mimetic Debio 1143 and ablative RT	Lung cancer	93
	Ablative RT	30 Gy × 1 F	Eliminating MDSCs	Combining anti-tumor CD8+ T cells and Ablative RT	Colon cancer	17
	Daily fractionated irradiation	3 Gy × 10 F	Don't changing MDSCs	-	Colon cancer	17
	SBRT	30 Gy × 1 F	Reducing MDSCs	Combining miR-21 targeting and SBRT	Lung cancer model	94
	photon RT	3 Gy × 8 F	Don't changing MDSCs but Increasing PD-L1	Combining photon RT with anti-PD-L1 immunotherapy	Colon tumor	96
Particle RT	proton radiation	-	Increasing MDSCs	-	Colon tumor	96
	proton radiation	-	The combination of RT and MSLN-targeting CAR T reducing MDSCs	Combining RT and MSLN-targeting CAR T	PDAC	97
	Carbon ion beams	5 GyE	Reducing MDSCs population	-	Melanoma	98
Radionuclide	^90^Y-NM600	-	Increasing MDSCs when ^90^Y-NM600 treatment followed by ADT	Combining the use of selective CXCR2 antagonist, reparixin, ^90^Y-NM600 and ADT treatment	Metastatic prostate cancer	101

F, Fractions;^90^Y-NM600, Yttrium-90-labeled NM600; Au@CAT, gold-catalase nanoaggregates; CRC, colorectal cancer; HNC, head and neck cancer; MNPs, magnetic nanoparticles; TMZ, temozolomide.

**Table 4:** Summary of therapeutics targeting MDSCs to synergize RT

MDSC- targeted strategy	Mechanism	Agent	Tumor type	RT schedule	Evidence level	Reference
Blocking the chemokine receptor	CCR5 antagonist	maraviroc	HCC	2Gy × 24 8Gy × 4	Preclinical (*in vivo*)	130
	CXCR2 antagonist	reparixin	Prostate cancer	^90^Y-NM600	Preclinical (*in vivo*)	101
	CXCR2 antagonist	SB225002	Colon carcinoma	5 Gy × 5 8 Gy × 3	Preclinical (*in vivo*)	88
	Multitarget tyrosine kinase inhibitor (VEGFR/PDGFR/FGFR)	Anlotinib	Lung cancer	8Gy × 3	Preclinical (*in vivo*)	137
	EP4 antagonist	ASP7657	TNBC	5 Gy × 3	Preclinical (*in vivo*)	139
	CCR2 blocking	Anti-CCR2 antibody	Colon carcinoma; Lung cancer	Local Ablative RT (20 Gy)	Preclinical (*in vivo*)	105
	Gr1 depletion	Anti-Gr1 antibody	Prostate cancer	^90^Y-NM600	Preclinical (*in vivo*)	101
	Gr1 depletion	Anti-Gr1 antibody	Bladder cancer	10 Gy × 2F	Preclinical (*in vivo*)	141
ICIs and immune agonists	PD-L1 blockade	Anti-PD-L1	Colorectal cancer	8 Gy × 3F	Preclinical (*in vivo*)	96
	PD-L1 blockade	Anti-PD-L1	HCC	Proton therapy	Preclinical (*in vivo*)	142
	VISTA blockade	Anti-VISTA antibody	HNC, breast cancer, and colorectal cancer	3 Gy × 5F/12 Gy × 2F	Preclinical (*in vivo*)	84
	VISTA blockade	Anti-VISTA antibody	TNBC	12 Gy × 2F	Preclinical (*in vivo*)	144
	VISTA blockade	Anti-VISTA antibody	NSCLC	20 Gy × 1F	Preclinical (*in vivo*)	145
	CAR T therapy	CAR T therapy	PDAC	Proton radiation therapy	Preclinical (*in vivo*)	97
	Immune agonist (IL-12 overexpression)	IL-12 plasmid	HCC	10 Gy × 1F	Preclinical (*in vivo*)	147
Restore metabolic homeostasis	CD73 blockade	Anti-CD73 antibody	Colorectal	8 Gy × 1F	Preclinical (*in vivo*)	82
	LXR agonists	GW3965 / RGX-104	NSCLC	10 Gy × 1F	Preclinical (*in vivo*)	152
Nanoparticle-based strategies	TLR7/8 agonist & PI3Kδ inhibitor co-delivery	PMNPs	TNBC	8 Gy × 3F	Preclinical (*in vivo*)	134
	ROS collection	Au@CAT	CRC	4 Gy × 1F	Preclinical (*in vivo*)	155
	Anti-CD47/PD-L1 & STING agonist	B-LNP	GBM	3 Gy × 3 F	Preclinical (*in vivo*)	118
	Repolarizing MDSCs	MNPs	GBM	2 Gy × 4 F	Preclinical (*in vivo*)	156
Other MDSC-targeted strategies	Cancer vaccine	FAPα-targeted vaccine	TNBC	8Gy × 3F	Preclinical (*in vivo*)	135
	Glutaminase inhibitor	CB839	HNSCC	3 Gy × 5F	Preclinical (*in vivo*)	103
	PDE5 inhibitor	Tadalafil	GBM	2 Gy × 5F	Preclinical (*in vivo*)	159
	SMAC mimetic	Debio 1143	NSCLC	30 Gy × 1F	Preclinical (*in vivo*)	93

**Table 5:** Completed clinical trials involving RT and targeting MDSCs

Target	Cancer type	Agent	Other interventions	Study phase	Key findings	References
CD73	Non-small Cell Lung Cancer	Oleclumab	Durvalumab, RT	I	No PRs or CRs associated with Oleclumab treatment	NCT03801902 (Last update posted: 2025-09)
COX2	Recurrent head and neck cancer	Celecoxib	Concurrent erlotinib and reirradiation	I	At a median follow-up of 11 months, the 1-year locoregional control, progression-free survival, and overall survival rates were 60%, 37%, and 55%, respectively.	184
IDO	Pediatric brain tumors	Indoximod	Temozolomide, palliative conformal radiation	I	Encouraging preliminary evidence of efficacy: Median overall survival was 13.3 months (n = 68, range 0.2-62.7); Four patients remain free of active disease longer than 36 months.	175
PDE5	GBM	Tadalafil	RT and temozolomide	Ib	The tadalafil cohort had a significantly lower ratio of circulating MDSCs than the control, but did not have significantly different PFS and OS than the historical control.	162
CXCR4	High grade glioma	Plerixafor	RT and temozolomide	I/II	Participants alive and without disease progression at 6 months after the start of the irradiation is from 83.3% to 100%.	NCT01977677 (Last update posted: 2018-10-23)
COX2	Non-Small Cell Lung Cancer	Celecoxib	thoracic RT	I/II	Overall survival rates at 1 and 2 years were 44.4% [95% confidence interval (CI), 21.6%-65.1%] and 22.2% (95% CI, 6.9%-42.9%), respectively. Progression-free survival at 1 year was 33.3% (95% CI, 13.7%-54.5%). The sample size was too small to draw conclusions regarding efficacy.	185
COX2	Cancer of the cervix	Celecoxib	Concurrent 5-fluorouracil and cisplatin chemotherapy and pelvic RT and brachytherapy	I/II	Limited clinical activity: At 2 years, the estimated PFS and OS rate was 69% and 83%, respectively.	170
IDO1	Lymphoma	Epacadostat	Intralesional SD-101 (Toll-receptor 9 Agonist), RT	I/II	Abscopal Response Rate (ARR) is 28.6%	NCT03322384 (Last update posted: 2022-02-01)
IDO	Primary Malignant Brain Tumors	Indoximod	Temozolomide, Stereotactic Radiation	I/II	No PRs or ORs	NCT02052648 (Last update posted: 2024-03-27)
CXCR4	High grade glioblastoma	Plerixafor	Whole brain radiation therapy with standard temozolomide chemo-RT	II	PFS at six months is 0.786 (0.598-1.0); Median survival is 398 days.	NCT03746080 (Last update posted: 2025-11-14)
COX2	Non-Small Cell Lung Cancer	Celecoxib	Cisplatin/Etoposide and Concurrent RT	II	Celecoxib did not improve survival	168
COX2	Esophageal cancer	Celecoxib	Irinotecan, cisplatin, concurrent radiation therapy	II	Celecoxib did not improve overall survival	169
COX2	Glioblastoma	Celecoxib	Standard radiation therapy, Temozolomide, thalidomide	II	The outcome did not meet the primary end point of improvement of 4-month PFS from study enrollment	167
IDO1	Recurrent Gliomas	Epacadostat	Bevacizumab, RT, Retifanlimab	II	Epacadostat did not improve survival and even compromised it.	NCT03532295 (Last update posted: 2025-11-21)
RAR	Cervical cancer	13-cis-retinoic acid (RA)	Interferon alpha-2b (IFN), radiation therapy	II	Treatment with RA-IFN-radiation did not demonstrate survival advantage over chemoradiation despite being less toxic.	180
Phosphatidylserine	GBM	Bavituximab	RT and temozolomide	II	The study met its primary endpoint with an OS-12 of 73% (95% confidence interval, 59%-90%). Post-treatment tumor specimens contained fewer immunosuppressive MDSCs.	158

For studies with no reference provided, data are from clinicaltrials.gov. ARR, Abscopal Response Rate; CI, Confidence Interval; COX2, Cyclooxygenase-2; CR, Complete Response; CSF1R, Colony-Stimulating Factor 1 Receptor; CXCR4, C-X-C Motif Chemokine Receptor 4; IDO, Indoleamine 2,3-Dioxygenase; IDO1, Indoleamine 2,3-Dioxygenase 1; IFN, Interferon; OS, Overall Survival; PFS, Progression-Free Survival; PR, Partial Response; RA, Retinoic Acid; RAR, Retinoic Acid Receptor.

**Table 6:** Clinical trials involving RT and targeting MDSCs (Ongoing/Results awaited)

ClinicalTrials.gov study identifier	Trial status	Cancer types	MDSC-related intervention	MDSC target	Other interventions	Study phase	Estimated study completion date
NCT04572451	Recruiting	Metastatic sold tumors	BMS-986253	IL-8	Nivolumab, SBRT	I	May-27
NCT04047706	Active, not recruiting	Glioblastoma	BMS-986205	IDO1	Nivolumab, RT, Temozolomide	I	Jun-27
NCT03516708	Recruiting	Rectal cancer	Epacadostat	IDO1	Preoperative Chemoradiation	I/II	Aug-30
NCT06439888	Recruiting	Oligo-metastatic solid cancer	ATRA (Vesanoid)	RAR	SBRT	I/II	Jan-27
NCT07015853	Not yet recruiting	TNBC	Axatilimab	CSF-1R	RT	II	Dec-32
NCT05491226	Recruiting	TNBC	Axatilimab	CSF-1R	Pembrolizumab, RT	II	Dec-26
NCT03875573	Active, not recruiting	Breast cancer	Oleclumab	CD73	Durvalumab, SBRT	II	Sep-29
NCT07146230	Active, not recruiting	Non-small cell lung cancer	Oleclumab	CD73	Durvalumab, RT	II	Feb-28
NCT05915442	Recruiting	Oligometastatic prostate cancer	AB680	CD73	SBRT, AB928, AB122	II	Dec-28
NCT07104266	Recruiting	Triple negative breast cancer	Celecoxib	COX2	RT	II	Aug-32
NCT07154316	Recruiting	Rectal cancer	Celecoxib	COX2	RT, serplulimab, capecitabine, oxaliplatin	II	Dec-28
NCT07150949	Recruiting	Rectal cancer	Celecoxib	COX2	RT, serplulimab, capecitabine, oxaliplatin	II	Aug-28
CT04049669	Recruiting	Relapsed brain tumors or newly diagnosed diffuse intrinsic pontine glioma (DIPG)	Indoximod	IDO	Chemotherapy and/or radiation	II	Oct-27
NCT06706401	Recruiting	Squamous cell carcinoma of the oropharynx, larynx or hypopharynx	ATRA (Vesanoid)	RAR	RT	III	Jan-29

AB680, CD73 Small Molecule Inhibitor; ARR, Abscopal Response Rate; ATRA, All-Trans Retinoic Acid; CI, Confidence Interval; COX2, Cyclooxygenase-2; CSF1R, Colony-Stimulating Factor 1 Receptor; CXCR4, C-X-C Motif Chemokine Receptor 4; DIPG, Diffuse Intrinsic Pontine Glioma; IDO, Indoleamine 2,3-Dioxygenase; IDO1, Indoleamine 2,3-Dioxygenase 1; IFN, Interferon; IL-8, Interleukin-8; PolyICLC, polyinosinic-polycytidylic acid stabilized with poly-L-lysine and carboxymethylcellulose; RA, Retinoic Acid; RAR, Retinoic Acid Receptor.

## Abbreviations

ADAM17: A Disintegrin and Metalloproteinase 17
ADT: androgen deprivation therapy
APCs: antigen-presenting cells
ARG1: arginase 1
ARR: abscopal response rate
ART: ablative RT
ATR: ataxia telangiectasia and Rad3
ATRA: all-trans retinoic acid
BAMBI: bone morphogenetic protein and activin membrane-bound inhibitor
bFGF: basic fibroblast growth factor
B-LNP: bridging-lipid nanoparticle
B-LNP: bridging-lipid nanoparticle
BNCT: boron neutron capture therapy
CAT-2B: cationic amino acid transporter
CFRT: conventional fractionated RT
CI: confidence interval
CMPs: common myeloid progenitors
COX2: cyclooxygenase-2
CR: complete response
CRT: calreticulin
CSF1R: colony-stimulating factor 1 receptor
CTL: cytotoxic T lymphocyte
CTLA-4: cytotoxic T lymphocyte-associated antigen 4
CXCR4: C-X-C Motif Chemokine Receptor 4
DAMPs: damage-associated molecular patterns
DCs: dendritic cells
DIPG: diffuse intrinsic pontine glioma
EBRT: external beam RT
eMDSCs: early-stage MDSCs
EMT: epithelial-mesenchymal transition
FAPα: fibroblast activation protein-alpha
FATP2: fatty acid transport protein 2
FGFR: fibroblast growth factor receptor
F-MDSCs: fibrocystic MDSCs
Gal-9: galectin-9
GBM: glioblastoma
GM-CSF: granulocyte-macrophage colony-stimulating factor
GMPs: granulocyte-macrophage progenitor
GPR81: G protein-coupled receptor 81
HMGB1: high mobility group box 1
HNSC: head and neck squamous cell carcinoma
HSCs: hematopoietic stem cells
HypoRT: Hypofractionated RT
ICD: immunogenic cell death
ICIs: immune checkpoint inhibitors
IDO: indoleamine 2,3-dioxygenase
IDO1: indoleamine 2,3-dioxygenase 1
IFN: interferons
IG-IMRT: image-guided intensity-modulated RT
IGRT: image-guided RT
IL-12: interleukin-12
IL-8: interleukin-8
IMCs: immature myeloid cells
IMRT: intensity-modulated RT
iNOS: inducible nitric oxide synthase
IRF8: interferon-related factor 8
LXRs: Liver-X nuclear receptors
m6A: N6-methyladenosine
MDSCs: myeloid-derived suppressor cells
MDPs: macrophage/dendritic cell progenitors
M-MDSCs: monocytic MDSCs
MMPs: matrix metalloproteinases
MSLN: mesothelin
NK cells: natural killer cells
NO: nitric oxide
NOX: NADPH oxidase
NSCLC: Non-Small Cell Lung Cancer
OS: overall survival
PD-1: programmed death 1
PDAC: pancreatic ductal adenocarcinoma
PDGFR: platelet derived growth factor receptor
PD-L1: programmed death-ligand 1
PFS: progression-free survival
PGE2: prostaglandin E2
PI3Kδ: PI3K delta
PMN-MDSCs: polymorphonuclear MDSCs
PolyICLC: polyinosinic-polycytidylic acid stabilized with poly-L-lysine and carboxymethylcellulose
PR: Partial Response
PT: proton therapy
RA: retinoic acid
RAR: retinoic acid receptor
RNS: reactive nitrogen species
ROS: reactive oxygen species
RT: radiotherapy
SABR: stereotactic ablative body irradiation
SABR: stereotactic ablative RT
SBRT: stereotactic body RT
SCF: stem cell factor
SMAC: secondary mitochondrial-derived activators of caspase
SRS: stereotactic radiosurgery
SRS/SBRT: Stereotactic RT
TAMs: tumor-associated macrophages
TANs: tumor-associated neutrophils
TCRs: T cell receptors
Th1: T-helper 1
TLR: Toll-like receptor
TLR4: Toll-like receptor 4
TME: tumor microenvironment
TNBC: triple-negative breast cancer
tmTNF-α: transmembrane TNF-α
Tregs: regulatory T cells
3D-CRT: 3D Conformal RT
TRT: targeted radionuclide therapy
VEGFR: vascular endothelial growth factor receptor
VISTA: V-domain Ig-containing suppressor of T-cell activation

## References

[1] Chandra RA, Keane FK, Voncken FEM (2021). Contemporary radiotherapy: present and future. The Lancet.

[2] Tang R, Yin J, Liu Y (2024). FLASH radiotherapy: A new milestone in the field of cancer radiotherapy. Cancer Lett.

[3] Zhou Z, Guan B, Xia H (2023). Particle radiotherapy in the era of radioimmunotherapy. Cancer Lett.

[4] Goldstein M, Kastan MB (2015). The DNA damage response: implications for tumor responses to radiation and chemotherapy. Annu Rev Med.

[5] Li Z, Lai X, Fu S (2022). Immunogenic cell death activates the tumor immune microenvironment to boost the immunotherapy efficiency. Adv Sci.

[6] Deng S, Wang J, Hu Y (2024). Irradiated tumour cell-derived microparticles upregulate MHC-I expression in cancer cells via DNA double-strand break repair pathway. Cancer Lett.

[7] Zhang C, Deng Z, Wu J (2024). HO-1 impairs the efficacy of radiotherapy by redistributing cGAS and STING in tumors. J Clin Invest.

[8] Ali Mohammad S, Hak A, Pogu SV (2023). Radiotherapy, photodynamic therapy, and cryoablation-induced abscopal effect: Challenges and future prospects. Cancer Innov.

[9] Wang L, Lynch C, Pitroda SP (2024). Radiotherapy and immunology. J Exp Med.

[10] Li K, Shi H, Zhang B (2021). Myeloid-derived suppressor cells as immunosuppressive regulators and therapeutic targets in cancer. Signal Transduct Target Ther.

[11] Li Y, Yang B, Miao H (2023). Nicotinamide N-methyltransferase promotes M2 macrophage polarization by IL6 and MDSC conversion by GM-CSF in gallbladder carcinoma. Hepatology.

[12] Wang Y, Liu H, Zhang Z (2023). G-MDSC-derived exosomes mediate the differentiation of M-MDSC into M2 macrophages promoting colitis-to-cancer transition. J Immunother Cancer.

[13] Pointer KB, Pitroda SP, Weichselbaum RR (2022). Radiotherapy and immunotherapy: open questions and future strategies. Trends Cancer.

[14] Cocito C, Branchtein M, Zhou XK (2025). Single-dose radiotherapy is more effective than fractionation when combined with anti-PD-1 immunotherapy in glioblastoma. Sci Rep.

[15] Ni H, Reitman ZJ, Zou W (2025). FLASH radiation reprograms lipid metabolism and macrophage immunity and sensitizes medulloblastoma to CAR-T cell therapy. Nat Cancer.

[16] Mao L, Zhou J-J, Xiao Y (2023). Immunogenic hypofractionated radiotherapy sensitising head and neck squamous cell carcinoma to anti-PD-L1 therapy in MDSC-dependent manner. Br J Cancer.

[17] Filatenkov A, Baker J, Mueller AMS (2015). Ablative Tumor Radiation Can Change the Tumor Immune Cell Microenvironment to Induce Durable Complete Remissions. Clin Cancer Res.

[18] Bortfeld T (2006). IMRT: a review and preview. Phys Med Biol.

[19] C S, Nair SS, Nagesh J (2025). Dosimetric comparison of 3D-conformal radiotherapy, volumetric modulated arc therapy, and h-VMAT in left breast radiotherapy under free-breathing and breath-hold conditions. Sci Rep.

[20] Jaffray DA (2023). A personal perspective on the development and future of cone-beam CT for image-guided radiotherapy. Med Phys.

[21] Malatesta T, Scaggion A, Giglioli FR (2023). Patient specific quality assurance in SBRT: a systematic review of measurement-based methods. Phys Med Biol.; 68. Epub ahead of print October 18.

[22] Schaub L, Harrabi SB, Debus J (2020). Particle therapy in the future of precision therapy. Br J Radiol.

[23] Song WY, Robar JL, Morén B (2021). Emerging technologies in brachytherapy. Phys Med Biol.

[24] Primac I, Tabury K, Tasdogan A (2025). The molecular blueprint of targeted radionuclide therapy. Nat Rev Clin Oncol.

[25] Guo Y, Hao S, Huang Q (2025). Unraveling the dual nature of FLASH radiotherapy: From normal tissue sparing to tumor control. Cancer Lett.

[26] Coghi P, Li J, Hosmane NS (2023). Next generation of boron neutron capture therapy (BNCT) agents for cancer treatment. Med Res Rev.

[27] Wang Y, Li Y, Yang Y (2024). In situ vaccination caused by diverse irradiation-driven cell death programs. Theranostics.

[28] Krieg AM (2025). New insights into the role of IFN-α/β and TLR7/8/9 in cancer immunotherapy and systemic autoimmunity. J Immunother Cancer.

[29] Gao Y, Yu B, Li L (2025). mtDNA/RNA boosts radiation-induced abscopal effect via M1 macrophage polarization-promoted IFN-β-dependent inflammatory response. Int Immunopharmacol.

[30] Gang X, Yan J, Li X (2024). Immune checkpoint inhibitors rechallenge in non-small cell lung cancer: Current evidence and future directions. Cancer Lett.

[31] Carlino MS, Larkin J, Long GV (2021). Immune checkpoint inhibitors in melanoma. Lancet Lond Engl.

[32] Antonia SJ, Villegas A, Daniel D (2017). Durvalumab after chemoradiotherapy in stage III non-small-cell lung cancer. N Engl J Med.

[33] Antonia SJ, Villegas A, Daniel D (2018). Overall survival with durvalumab after chemoradiotherapy in stage III NSCLC. N Engl J Med.

[34] Rahma OE, Yothers G, Hong TS (2021). Use of Total Neoadjuvant Therapy for Locally Advanced Rectal Cancer: Initial Results From the Pembrolizumab Arm of a Phase 2 Randomized Clinical Trial. JAMA Oncol.

[35] Lee NY, Ferris RL, Psyrri A (2021). Avelumab plus standard-of-care chemoradiotherapy versus chemoradiotherapy alone in patients with locally advanced squamous cell carcinoma of the head and neck: a randomised, double-blind, placebo-controlled, multicentre, phase 3 trial. Lancet Oncol.

[36] Machiels J-P, Tao Y, Burtness B (2022). LBA5 Primary results of the phase III KEYNOTE-412 study: Pembrolizumab (pembro) with chemoradiation therapy (CRT) vs placebo plus CRT for locally advanced (LA) head and neck squamous cell carcinoma (HNSCC). Ann Oncol.

[37] Lasser SA, Ozbay Kurt FG, Arkhypov I (2024). Myeloid-derived suppressor cells in cancer and cancer therapy. Nat Rev Clin Oncol.

[38] Lee HJ, Choi YR, Ko JH (2024). Defining mesenchymal stem/stromal cell-induced myeloid-derived suppressor cells using single-cell transcriptomics. Mol Ther.

[39] Dey S, Mondal A, DuHadaway JB (2021). IDO1 Signaling through GCN2 in a Subpopulation of Gr-1+ Cells Shifts the IFNγ/IL6 Balance to Promote Neovascularization. Cancer Immunol Res.

[40] Veglia F, Sanseviero E, Gabrilovich DI (2021). Myeloid-derived suppressor cells in the era of increasing myeloid cell diversity. Nat Rev Immunol.

[41] Bronte V, Brandau S, Chen S-H (2016). Recommendations for myeloid-derived suppressor cell nomenclature and characterization standards. Nat Commun.

[42] Cortez-Retamozo V, Etzrodt M, Newton A (2012). Origins of tumor-associated macrophages and neutrophils. Proc Natl Acad Sci U S A.

[43] Karin N (2020). The development and homing of myeloid-derived suppressor cells: from a two-stage model to a multistep narrative. Front Immunol.

[44] Mouchemore KA, Anderson RL (2021). Immunomodulatory effects of G-CSF in cancer: Therapeutic implications. Semin Immunol.

[45] Li W, Zhang X, Chen Y (2016). G-CSF is a key modulator of MDSC and could be a potential therapeutic target in colitis-associated colorectal cancers. Protein Cell.

[46] Que H, Fu Q, Lan T (2022). Tumor-associated neutrophils and neutrophil-targeted cancer therapies. Biochim Biophys Acta BBA - Rev Cancer.

[47] Wang J-C, Sun L (2022). PD-1/PD-L1, MDSC Pathways, and Checkpoint Inhibitor Therapy in Ph(-) Myeloproliferative Neoplasm: A Review. Int J Mol Sci.

[48] Noman MZ, Desantis G, Janji B (2014). PD-L1 is a novel direct target of HIF-1α, and its blockade under hypoxia enhanced MDSC-mediated T cell activation. J Exp Med.

[49] Jeong H, Koh J, Kim S (2025). Cell-intrinsic PD-L1 signaling drives immunosuppression by myeloid-derived suppressor cells through IL-6/Jak/Stat3 in PD-L1-high lung cancer. J Immunother Cancer.

[50] Yaseen MM, Abuharfeil NM, Darmani H (2020). Mechanisms of immune suppression by myeloid-derived suppressor cells: the role of interleukin-10 as a key immunoregulatory cytokine. Open Biol.

[51] Batlle E, Massagué J (2019). Transforming growth factor-β signaling in immunity and cancer. Immunity.

[52] Siret C, Collignon A, Silvy F (2020). Deciphering the crosstalk between myeloid-derived suppressor cells and regulatory T cells in pancreatic ductal adenocarcinoma. Front Immunol.

[53] Peng D, Fu M, Wang M (2022). Targeting TGF-β signal transduction for fibrosis and cancer therapy. Mol Cancer.

[54] Karadima E, Chavakis T, Alexaki VI (2025). Arginine metabolism in myeloid cells in health and disease. Semin Immunopathol.

[55] Bozkus CC, Elzey BD, Crist SA (2015). Expression of Cationic Amino Acid Transporter 2 (CAT2) Is Required for Myeloid Derived Suppressor Cell-Mediated Control of T Cell Immunity. J Immunol Baltim Md 1950.

[56] Srivastava MK, Sinha P, Clements VK (2010). Myeloid-derived suppressor cells inhibit T-cell activation by depleting cystine and cysteine. Cancer Res.

[57] Fujiwara Y, Kato S, Nesline MK (2022). Indoleamine 2,3-dioxygenase (IDO) inhibitors and cancer immunotherapy. Cancer Treat Rev.

[58] Li J, Wang L, Chen X (2017). CD39/CD73 upregulation on myeloid-derived suppressor cells via TGF-β-mTOR-HIF-1 signaling in patients with non-small cell lung cancer. Oncoimmunology.

[59] Allard B, Allard D, Buisseret L (2020). The adenosine pathway in immuno-oncology. Nat Rev Clin Oncol.

[60] Liu X, Ding Q, Zhang H (2025). The CD39-CD73-adenosine axis: Master regulator of immune evasion and therapeutic target in pancreatic ductal adenocarcinoma. Biochim Biophys Acta BBA - Rev Cancer.

[61] Cozzolino M, Gyöngyösi A, Korpos E (2023). The Voltage-Gated Hv1 H+ Channel Is Expressed in Tumor-Infiltrating Myeloid-Derived Suppressor Cells. Int J Mol Sci.

[62] Adeshakin AO, Liu W, Adeshakin FO (2021). Regulation of ROS in myeloid-derived suppressor cells through targeting fatty acid transport protein 2 enhanced anti-PD-L1 tumor immunotherapy. Cell Immunol.

[63] Huang J, Zhao Y, Zhao K (2023). Function of reactive oxygen species in myeloid-derived suppressor cells. Front Immunol.

[64] Parker K, Sinha P, Horn LA (2014). HMGB1 enhances immune suppression by facilitating the differentiation and suppressive activity of myeloid-derived suppressor cells. Cancer Res.

[65] Gehad A, Lichtman M, Schmults C (2012). Nitric oxide-producing myeloid-derived suppressor cells inhibit vascular E- selectin expression in human squamous cell carcinomas. J Invest Dermatol.

[66] Wang H, Zhou F, Qin W (2025). Metabolic regulation of myeloid-derived suppressor cells in tumor immune microenvironment: Targets and therapeutic strategies. Theranostics.

[67] Yang Y, Li C, Liu T (2020). Myeloid-Derived Suppressor Cells in Tumors: From Mechanisms to Antigen Specificity and Microenvironmental Regulation. Front Immunol.

[68] Zhang H, Li Z, Wang L (2017). Critical role of myeloid-derived suppressor cells in tumor-induced liver immune suppression through inhibition of NKT cell function. Front Immunol.

[69] Shen M, Wang J, Yu W (2018). A novel MDSC-induced PD-1- PD-L1+ B-cell subset in breast tumor microenvironment possesses immuno-suppressive properties. OncoImmunology.

[70] Vetsika E-K, Koukos A, Kotsakis A (2019). Myeloid-derived suppressor cells: Major figures that shape the immunosuppressive and angiogenic network in cancer. Cells.

[71] Jiang T, Zhu X, Yin Z (2025). Dual role of baimao-longdan-congrong-fang in inhibiting staphylococcus aureus virulence factors and regulating TNF-α/TNFR1/NF-κB/MMP9 axis. Phytomedicine Int J Phytother Phytopharm.

[72] Shojaei F, Wu X, Zhong C (2007). Bv8 regulates myeloid-cell-dependent tumour angiogenesis. Nature.

[73] Li C, Xing X, Li M (2025). Bile acids produced by gut microbiota activate TGR5 to promote colorectal liver metastasis progression by inducing MDSCs infiltration in liver. Int Immunopharmacol.

[74] Yang L, DeBusk LM, Fukuda K (2004). Expansion of myeloid immune suppressor gr+CD11b+ cells in tumor-bearing host directly promotes tumor angiogenesis. Cancer Cell.

[75] Ouzounova M, Lee E, Piranlioglu R (2017). Monocytic and granulocytic myeloid derived suppressor cells differentially regulate spatiotemporal tumour plasticity during metastatic cascade. Nat Commun.

[76] Cole K, Pravoverov K, Talmadge JE (2021). Role of myeloid-derived suppressor cells in metastasis. Cancer Metastasis Rev.

[77] Sprouse ML, Welte T, Boral D (2019). PMN-MDSCs enhance CTC metastatic properties through reciprocal interactions via ROS/notch/nodal signaling. Int J Mol Sci.

[78] Hiratsuka S, Watanabe A, Sakurai Y (2008). The S100A8-serum amyloid A3-TLR4 paracrine cascade establishes a pre-metastatic phase. Nat Cell Biol.

[79] Shi H, Zhang J, Han X (2017). Recruited monocytic myeloid-derived suppressor cells promote the arrest of tumor cells in the premetastatic niche through an IL-1β-mediated increase in E-selectin expression. Int J Cancer.

[80] Lee S-E, Lim J-Y, Kim TW (2018). Matrix metalloproteinase-9 in monocytic myeloid-derived suppressor cells correlate with early infections and clinical outcomes in allogeneic hematopoietic stem cell transplantation. Biol Blood Marrow Transplant.

[81] Jiménez-Cortegana C, Galassi C, Klapp V (2022). Myeloid-derived suppressor cells and radiotherapy. Cancer Immunol Res.

[82] An R, Wu C, Tang C (2024). Blockade of CD73 potentiates radiotherapy antitumor immunity and abscopal effects via STING pathway. Cell Death Discov.

[83] Zhang Z, Yao Z, Zhang Z (2023). Local radiotherapy for murine breast cancer increases risk of metastasis by promoting the recruitment of M-MDSCs in lung. Cancer Cell Int.

[84] Nambiar DK, Maddineni S, Langthasa J (2025). VISTA immune checkpoint blunts radiotherapy-induced antitumor immune response. Cell Rep.

[85] Hu S, Zhan N, Li J (2025). RBM15 recruits myeloid-derived suppressor cells via the m6A-IGF2BP3/CBR3-AS1/miR-409-3p/CXCL1 axis, facilitating radioresistance in non-small-cell lung cancer. J Transl Med.

[86] De Bari B, Porta L, Mazzola R (2016). Hypofractionated radiotherapy in pancreatic cancer: Lessons from the past in the era of stereotactic body radiation therapy. Crit Rev Oncol Hematol.

[87] Picardi C, Perret I, Miralbell R (2018). Hypofractionated radiotherapy for prostate cancer in the postoperative setting: What is the evidence so far?. Cancer Treat Rev.

[88] Zhang B, Hu M, Ma Q (2023). Optimized CAR-T therapy based on spatiotemporal changes and chemotactic mechanisms of MDSCs induced by hypofractionated radiotherapy. Mol Ther.

[89] Lin L, Kane N, Kobayashi N (2021). High-dose per Fraction Radiotherapy Induces Both Antitumor Immunity and Immunosuppressive Responses in Prostate Tumors. Clin Cancer Res.

[90] Chen J, Wang Z, Ding Y (2020). Hypofractionated Irradiation Suppressed the Off-Target Mouse Hepatocarcinoma Growth by Inhibiting Myeloid-Derived Suppressor Cell-Mediated Immune Suppression. Front Oncol.

[91] Teng F, Yin T, Ju X (2023). Optimum fractionation of radiation to combine PD-1 blockade. MedComm.

[92] Goodman KA, Wiegner EA, Maturen KE (2010). Dose-escalation study of single-fraction stereotactic body radiotherapy for liver malignancies. Int J Radiat Oncol.

[93] Tao Z, McCall NS, Wiedemann N (2019). SMAC Mimetic Debio 1143 and Ablative Radiation Therapy Synergize to Enhance Antitumor Immunity against Lung Cancer. Clin Cancer Res.

[94] Zhao C, Tang Q, Yang C (2024). Stereotactic body radiation therapy suppresses myeloid-derived suppressor cells by regulating miR-21/sorbin and SH3 domain-containing protein 1 axis. Hum Exp Toxicol.

[95] Stewart RD, Carlson DJ, Butkus MP (2018). A comparison of mechanism-inspired models for particle relative biological effectiveness (RBE). Med Phys.

[96] Burckel H, Nicol A, Mura C (2025). Distinct immune responses to proton and photon radiotherapy: implications for anti-PD-L1 combination therapy in colorectal cancer. J Transl Med.

[97] Amit U, Uslu U, Verginadis II (2024). Proton radiation boosts the efficacy of mesothelin-targeting chimeric antigen receptor T cell therapy in pancreatic cancer. Proc Natl Acad Sci.

[98] Zhou H, Yang P, Li H (2021). Carbon ion radiotherapy boosts anti-tumour immune responses by inhibiting myeloid-derived suppressor cells in melanoma-bearing mice. Cell Death Discov.

[99] Chang C-H, Chen F-H, Wang L-W (2024). Circulating M-MDSC Levels as an Assessment Marker for Post-Treatment Tumor Progression in Recurrent HNC Patients Following Radiation Therapy: A Case Series. J Clin Med.

[100] Sgouros G, Bodei L, McDevitt MR (2020). Radiopharmaceutical therapy in cancer: clinical advances and challenges. Nat Rev Drug Discov.

[101] Muralidhar A, Hernandez R, Morris ZS (2024). Myeloid-derived suppressor cells attenuate the antitumor efficacy of radiopharmaceutical therapy using90 Y-NM600 in combination with androgen deprivation therapy in murine prostate tumors. J Immunother Cancer.

[102] He S, Zheng L, Qi C (2025). Myeloid-derived suppressor cells (MDSCs) in the tumor microenvironment and their targeting in cancer therapy. Mol Cancer.

[103] Guan L, Nambiar DK, Cao H (2023). NFE2L2 mutations enhance radioresistance in head and neck cancer by modulating intratumoral myeloid cells. Cancer Res.

[104] Hou Y, Yang K, Wang L (2024). Radiotherapy enhances metastasis through immune suppression by inducing PD-L1 and MDSC in distal sites. Clin Cancer Res.

[105] Liang H, Deng L, Hou Y (2017). Host STING-dependent MDSC mobilization drives extrinsic radiation resistance. Nat Commun.

[106] Iwata T, Kondo Y, Kimura O (2016). PD-L1+MDSCs are increased in HCC patients and induced by soluble factor in the tumor microenvironment. Sci Rep.

[107] Duraiswamy J, Freeman GJ, Coukos G (2013). Therapeutic PD-1 Pathway Blockade Augments with other Modalities of Immunotherapy to Prevent Immune Decline in Ovarian Cancer. Cancer Res.

[108] Gabrilovich DI, Nagaraj S (2009). Myeloid-derived-suppressor cells as regulators of the immune system. Nat Rev Immunol.

[109] Chen L, Shi V, Wang S (2023). SCCA1/SERPINB3 suppresses antitumor immunity and blunts therapy-induced T cell responses via STAT-dependent chemokine production. J Clin Invest.

[110] Oweida AJ, Mueller AC, Piper M (2021). Response to radiotherapy in pancreatic ductal adenocarcinoma is enhanced by inhibition of myeloid-derived suppressor cells using STAT3 anti-sense oligonucleotide. Cancer Immunol Immunother.

[111] Liu J, Lin W-P, Su W (2023). Sunitinib attenuates reactive MDSCs enhancing anti-tumor immunity in HNSCC. Int Immunopharmacol.

[112] Kobayashi Y, Yamada D, Kawai T (2020). Different immunological effects of the molecular targeted agents sunitinib, everolimus and temsirolimus in patients with renal cell carcinoma. Int J Oncol.

[113] Duan XL, Guo JP, Li F (2020). Sunitinib inhibits PD-L1 expression in osteosarcoma by targeting STAT3 and remodels the immune system in tumor-bearing mice. Future Oncol Lond Engl.

[114] Chen H-M, Ma G, Gildener-Leapman N (2015). Myeloid-Derived Suppressor Cells as an Immune Parameter in Patients with Concurrent Sunitinib and Stereotactic Body Radiotherapy. Clin Cancer Res.

[115] Chen C, Xu P (2023). Cellular functions of cGAS-STING signaling. Trends Cell Biol.

[116] Samson N, Ablasser A (2022). The cGAS-STING pathway and cancer. Nat Cancer.

[117] Jiang Q, Chen Z, Jiang J (2025). The role of cGAS-STING in remodeling the tumor immune microenvironment induced by radiotherapy. Crit Rev Oncol Hematol.

[118] Zhang P, Rashidi A, Zhao J (2023). STING agonist-loaded, CD47/PD-L1-targeting nanoparticles potentiate antitumor immunity and radiotherapy for glioblastoma. Nat Commun.

[119] Liu J, Xiao Q, Xiao J (2022). Wnt/β-catenin signalling: function, biological mechanisms, and therapeutic opportunities. Signal Transduct Target Ther.

[120] Jiang X, Liu B, Nie Z (2021). The role of m6A modification in the biological functions and diseases. Signal Transduct Target Ther.

[121] Mao H, Zhao X, Sun S (2025). NF-κB in inflammation and cancer. Cell Mol Immunol.

[122] Shi X, Yang J, Deng S (2022). TGF-β signaling in the tumor metabolic microenvironment and targeted therapies. J Hematol OncolJ Hematol Oncol.

[123] Wang L, Dou X, Chen S (2023). YTHDF2 inhibition potentiates radiotherapy antitumor efficacy. Cancer Cell.

[124] Wang L, Si W, Yu X (2023). Epitranscriptional regulation of TGF-β pseudoreceptor BAMBI by m6A/YTHDF2 drives extrinsic radioresistance. J Clin Invest.

[125] Jayaraman P, Parikh F, Newton JM (2018). TGF-β1 programmed myeloid-derived suppressor cells (MDSC) acquire immune-stimulating and tumor killing activity capable of rejecting established tumors in combination with radiotherapy. OncoImmunology.

[126] Ye X, Liu J, Quan R (2023). DKK1 affects survival of patients with head and neck squamous cell carcinoma by inducing resistance to radiotherapy and immunotherapy. Radiother Oncol.

[127] Zhang Md J, Zhang Md L, Yang Md Y (2021). Polymorphonuclear-MDSCs facilitate tumor regrowth after radiation by suppressing CD8+ T cells. Int J Radiat Oncol.

[128] Yang X, Lu Y, Hang J (2020). Lactate-modulated immunosuppression of myeloid-derived suppressor cells contributes to the radioresistance of pancreatic cancer. Cancer Immunol Res.

[129] Chen M-F, Chen Y-Y, Chen W-C (2023). The relationship of nutritional status with anticancer immunity and its prognostic value for head and neck cancer. Mol Carcinog.

[130] Chen J, Lin Q, Lan R (2025). A CCR5 antagonist enhances the radiosensitivity of hepatocarcinoma in a mouse model. J Radiat Res (Tokyo).

[131] Napolitano M, D'Alterio C, Cardone E (2015). Peripheral myeloid-derived suppressor and T regulatory PD-1 positive cells predict response to neoadjuvant short-course radiotherapy in rectal cancer patients. Oncotarget.

[132] Haist M, Stege H, Grabbe S (2021). The functional crosstalk between myeloid-derived suppressor cells and regulatory T cells within the immunosuppressive tumor microenvironment. Cancers.

[133] Park S-Y, Pylaeva E, Bhuria V (2025). Harnessing myeloid cells in cancer. Mol Cancer.

[134] Yazdimamaghani M, Kolupaev OV, Lim C (2025). Tumor microenvironment immunomodulation by nanoformulated TLR 7/8 agonist and PI3k delta inhibitor enhances therapeutic benefits of radiotherapy. Biomaterials.

[135] Chen M, Xiao L, Jia H (2023). Stereotactic ablative radiotherapy and FAPα-based cancer vaccine suppresses metastatic tumor growth in 4T1 mouse breast cancer. Radiother Oncol.

[136] Yu Y, Wang Y, Zhang J (2025). Anaerobic probiotics-in situ Se nanoradiosensitizers selectively anchor to tumor with immuno-regulations for robust cancer radio-immunotherapy. Biomaterials.

[137] Jiang Y, Qiao S, Li L (2024). Combination of radiotherapy and Anlotinib enhances benefit from immunotherapy to liver metastasis and abscopal tumor from lung cancer. Int Immunopharmacol.

[138] Obermajer N, Muthuswamy R, Lesnock J (2011). Positive feedback between PGE2 and COX2 redirects the differentiation of human dendritic cells toward stable myeloid-derived suppressor cells. Blood.

[139] Nishibata T, Amino N, Tanaka-Kado R (2023). Blockade of EP4 by ASP7657 Modulates Myeloid Cell Differentiation In Vivo and Enhances the Antitumor Effect of Radiotherapy. BioMed Res Int.

[140] Guo S, Zhang Q, Guo Y (2025). The role and therapeutic targeting of the CCL2/CCR2 signaling axis in inflammatory and fibrotic diseases. Front Immunol.

[141] Yamamoto S, Kato M, Takeyama Y (2023). Irradiation plus myeloid-derived suppressor cell-targeted therapy for overcoming treatment resistance in immunologically cold urothelial carcinoma. Br J Cancer.

[142] Chen M-F, Chen P-T, Hsieh C-C (2023). Effect of Proton Therapy on Tumor Cell Killing and Immune Microenvironment for Hepatocellular Carcinoma. Cells.

[143] Zhang RJ, Kim TK (2024). VISTA-mediated immune evasion in cancer. Exp Mol Med.

[144] Pilones KA, Hensler M, Daviaud C (2020). Converging focal radiation and immunotherapy in a preclinical model of triple negative breast cancer: contribution of VISTA blockade. OncoImmunology.

[145] Zhang Y, Hu J, Ji K (2023). CD39 inhibition and VISTA blockade may overcome radiotherapy resistance by targeting exhausted CD8+ T cells and immunosuppressive myeloid cells. Cell Rep Med.

[146] Del Vecchio M, Bajetta E, Canova S (2007). Interleukin-12: biological properties and clinical application. Clin Cancer Res.

[147] Wu C-J, Tsai Y-T, Lee I-J (2018). Combination of radiation and interleukin 12 eradicates large orthotopic hepatocellular carcinoma through immunomodulation of tumor microenvironment. OncoImmunology.

[148] Xia C, Yin S, To KKW (2023). CD39/CD73/A2AR pathway and cancer immunotherapy. Mol Cancer.

[149] Yan D, Yang Q, Shi M (2013). Polyunsaturated fatty acids promote the expansion of myeloid-derived suppressor cells by activating the JAK / STAT 3 pathway. Eur J Immunol.

[150] Bensinger SJ, Bradley MN, Joseph SB (2008). LXR signaling couples sterol metabolism to proliferation in the acquired immune response. Cell.

[151] Tavazoie MF, Pollack I, Tanqueco R (2018). LXR/ApoE activation restricts innate immune suppression in cancer. Cell.

[152] Liang H, Shen X (2020). LXR activation radiosensitizes non-small cell lung cancer by restricting myeloid-derived suppressor cells. Biochem Biophys Res Commun.

[153] Herbst RS, Majem M, Barlesi F (2022). COAST: An Open-Label, Phase II, Multidrug Platform Study of Durvalumab Alone or in Combination with Oleclumab or Monalizumab in Patients with Unresectable, Stage III Non-Small-Cell Lung Cancer. J Clin Oncol.

[154] Kuboki Y, Koyama T, Matsubara N (2024). PD -1 inhibition with retifanlimab and/or arginase inhibition with INCB001158 in Japanese patients with solid tumors: A phase I study. Cancer Med.

[155] Najafi A, Keykhaee M, Kazemi MH (2023). Catalase-gold nanoaggregates manipulate the tumor microenvironment and enhance the effect of low-dose radiation therapy by reducing hypoxia. Biomed Pharmacother.

[156] Wu C, Muroski ME, Miska J (2019). Repolarization of myeloid derived suppressor cells via magnetic nanoparticles to promote radiotherapy for glioma treatment. Nanomedicine Nanotechnol Biol Med.

[157] Stasi I, Cappuzzo F (2014). Profile of bavituximab and its potential in the treatment of non-small-cell lung cancer. Lung Cancer Auckl NZ.

[158] Ly KI, Richardson LG, Liu M (2023). Bavituximab Decreases Immunosuppressive Myeloid-Derived Suppressor Cells in Newly Diagnosed Glioblastoma Patients. Clin Cancer Res.

[159] Ghosh S, Huang J, Inkman M (2023). Role of radiation-induced circulating myeloid-derived suppressor cells on systemic lymphopenia after chemoradiotherapy for glioblastoma. Sci Transl Med.

[160] Galiè N, Brundage BH, Ghofrani HA (2009). Tadalafil therapy for pulmonary arterial hypertension. Circulation.

[161] Porst H, Giuliano F, Glina S (2006). Evaluation of the efficacy and safety of once-a-day dosing of tadalafil 5mg and 10mg in the treatment of erectile dysfunction: results of a multicenter, randomized, double-blind, placebo-controlled trial. Eur Urol.

[162] Ghosh S, Johanns TM, Chheda MG (2023). A pilot phase Ib study to evaluate tadalafil to overcome immunosuppression during chemoradiotherapy for IDH-wild-type glioblastoma. Neuro-Oncol Adv.

[163] Lynch C, Pitroda SP, Weichselbaum RR (2024). Radiotherapy, immunity, and immune checkpoint inhibitors. Lancet Oncol.

[164] Prima V, Kaliberova LN, Kaliberov S (2017). COX2/mPGES1/PGE2 pathway regulates PD-L1 expression in tumor-associated macrophages and myeloid-derived suppressor cells. Proc Natl Acad Sci.

[165] Jahani V, Yazdani M, Badiee A (2023). Liposomal celecoxib combined with dendritic cell therapy enhances antitumor efficacy in melanoma. J Controlled Release.

[166] Meyerhardt JA, Shi Q, Fuchs CS (2021). Effect of celecoxib vs placebo added to standard adjuvant therapy on disease-free survival among patients with stage III colon cancer: the CALGB/SWOG 80702 (alliance) randomized clinical trial. JAMA.

[167] Kesari S, Schiff D, Henson JW (2008). Phase II study of temozolomide, thalidomide, and celecoxib for newly diagnosed glioblastoma in adults. Neuro-Oncol.

[168] Bi N, Liang J, Zhou Z (2019). Effect of Concurrent Chemoradiation with Celecoxib vs Concurrent Chemoradiation Alone on Survival Among Patients with Non-Small Cell Lung Cancer with and Without Cyclooxygenase 2 Genetic Variants: A Phase 2 Randomized Clinical Trial. JAMA Netw Open.

[169] Cleary JM, Mamon HJ, Szymonifka J (2016). Neoadjuvant irinotecan, cisplatin, and concurrent radiation therapy with celecoxib for patients with locally advanced esophageal cancer. BMC Cancer.

[170] Gaffney DK, Winter K, Dicker AP (2007). Efficacy and patterns of failure for locally advanced cancer of the cervix treated with celebrex (celecoxib) and chemoradiotherapy in RTOG 0128. Int J Radiat Oncol Biol Phys.

[171] Sun R, Luo H, Su J (2021). Olaparib Suppresses MDSC Recruitment via SDF1α/CXCR4 Axis to Improve the Anti-tumor Efficacy of CAR-T Cells on Breast Cancer in Mice. Mol Ther J Am Soc Gene Ther.

[172] Schaff LR, Mellinghoff IK (2023). Glioblastoma and Other Primary Brain Malignancies in Adults: A Review. JAMA.

[173] Holmgaard RB, Zamarin D, Li Y (2015). Tumor-Expressed IDO Recruits and Activates MDSCs in a Treg-Dependent Manner. Cell Rep.

[174] Long GV, Dummer R, Hamid O (2019). Epacadostat plus pembrolizumab versus placebo plus pembrolizumab in patients with unresectable or metastatic melanoma (ECHO-301/KEYNOTE-252): a phase 3, randomised, double-blind study. Lancet Oncol.

[175] Johnson TS, MacDonald TJ, Pacholczyk R (2024). Indoximod-based chemo-immunotherapy for pediatric brain tumors: A first-in-children phase I trial. Neuro-Oncol.

[176] Nefedova Y, Fishman M, Sherman S (2007). Mechanism of all-trans retinoic acid effect on tumor-associated myeloid-derived suppressor cells. Cancer Res.

[177] Tobin RP, Cogswell DT, Cates VM (2023). Targeting MDSC Differentiation Using ATRA: A Phase I/II Clinical Trial Combining Pembrolizumab and All-Trans Retinoic Acid for Metastatic Melanoma. Clin Cancer Res Off J Am Assoc Cancer Res.

[178] Iclozan C, Antonia S, Chiappori A (2013). Therapeutic regulation of myeloid-derived suppressor cells and immune response to cancer vaccine in patients with extensive stage small cell lung cancer. Cancer Immunol Immunother CII.

[179] Tobin RP, Jordan KR, Robinson WA (2018). Targeting myeloid-derived suppressor cells using all-trans retinoic acid in melanoma patients treated with Ipilimumab. Int Immunopharmacol.

[180] Basu P, Jenson AB, Majhi T (2016). Phase 2 randomized controlled trial of radiation therapy plus concurrent interferon-alpha and retinoic acid versus cisplatin for stage III cervical carcinoma. Int J Radiat Oncol.

[181] Tomassetti C, Insinga G, Gimigliano F (2024). Insights into CSF-1R Expression in the Tumor Microenvironment. Biomedicines.

[182] King RJ, Shukla SK, He C (2022). CD73 induces GM-CSF/MDSC-mediated suppression of T cells to accelerate pancreatic cancer pathogenesis. Oncogene.

[183] Kwong TT, Xiong Z, Zhang Y (2025). Overcoming immunotherapy resistance in hepatocellular carcinoma by targeting myeloid IL-8/CXCR2 signaling. Mol Ther J Am Soc Gene Ther.

[184] Kao J, Genden EM, Chen C-T (2011). Phase 1 trial of concurrent erlotinib, celecoxib, and reirradiation for recurrent head and neck cancer. Cancer.

[185] Gore E, Bae K, Langer C (2011). Phase I/II trial of a COX-2 inhibitor with limited field radiation for intermediate prognosis patients who have locally advanced non-small-cell lung cancer: radiation therapy oncology group 0213. Clin Lung Cancer.

